# Sodium tanshinone IIA sulfonate alleviates osteoarthritis through targeting SIRT1

**DOI:** 10.1186/s13020-025-01166-2

**Published:** 2025-09-01

**Authors:** Mao Xu, Xulei Sun, Xiao Ma, Zixuan Qin, Xin Gao, Xinxin Jin, Hongzhi Sun

**Affiliations:** 1 Department of Physiology and Pathophysiology, School of Basic Medical Sciences, Xi’an Jiaotong University Health Science Center, Xi’an, Shaanxi 710061 People’s Republic of China; 2 Department of Pharmacy, Hubei Cancer Hospital, Tongji Medical College, Huazhong University of Science and Technology, Wuhan, 430079 People’s Republic of China; 3 Center for Translational Medicine, The First Affiliated Hospital of Xi’an Jiaotong University, Xi’an, Shaanxi 710061 People’s Republic of China

**Keywords:** Osteoarthritis, SIRT1, Sodium tanshinone IIA sulfonate, NF-κB p65, Inflammation

## Abstract

**Background:**

Osteoarthritis (OA), a chronic degenerative disease, is characterized by the loss of articular cartilage, impacting more than 500 million individuals worldwide. Sodium tanshinone IIA sulfonate (STS) is a water-soluble derivative of tanshinone IIA derived from Salvia miltiorrhiza and has anti-inflammatory and anti-oxidative functions. Although STS shows significant pharmacological effects and mechanisms in treating various diseases in vivo and in vitro, its specific treatments and mechanisms for OA remain largely unknown.

**Materials and methods:**

Primary chondrocytes were stimulated with interleukin-1β (IL-1β) to establish an in vitro OA model. The optimal concentration of STS for application on chondrocytes was determined to be 100 μM using MTT assays. The effects of STS on catabolic gene expression were assessed through real-time quantitative PCR (RT-qPCR). Western blotting, immunoprecipitation (IP), and mutation techniques were employed to investigate the impact of STS on the deacetylation of nuclear factor kappa B subunit p65 (NF-κB p65) at Lys310 by silent information regulator 1 (SIRT1). Furthermore, RT-qPCR, Enzyme-linked immunosorbent assay (ELISA), transmission electron microscopy, and immunohistochemistry staining were utilized to elucidate the molecular mechanisms underlying NF-κB-driven inflammation and ferroptosis. The destabilization of the medial meniscus (DMM) surgery-induced OA mouse model was employed to evaluate the therapeutic potential of STS in OA treatment. Safranin O-fast green and hematoxylin and eosin (HE) staining analyses were conducted to assess the impact of STS on OA. Additionally, tamoxifen (TM)-inducible *Sirt1* cartilage-specific conditional knockout (*Sirt1*cKO) mice were utilized to further validate the effects of STS on OA.

**Results:**

STS suppressed the gene expression levels of collagen type X alpha 1 (COL10A1), matrix metalloproteinase-13 (MMP13), and Caspase3, thereby mitigating matrix degradation and apoptosis in IL-1β-induced primary chondrocytes. Additionally, STS enhanced the expression of SIRT1 in these cells. Furthermore, STS facilitated the deacetylation of NF-κB p65 at Lysine (K) 310 by SIRT1 in primary chondrocytes. STS also inhibited NF-κB p65-mediated inflammation and ferroptosis, contributing to the amelioration of OA. In the DMM surgery-induced OA mice model, STS mitigated OA phenotypes by inhibiting matrix degradation and apoptosis, facilitating SIRT1-mediated deacetylation of NF-κB p65, and subsequently suppressing NF-κB p65-driven inflammation and ferroptosis. Finally, the use of *Sirt1*cKO transgenic mice further confirmed the effects of STS in attenuating OA progression.

**Conclusion:**

STS ameliorated OA by activating SIRT1 and inhibiting NF-κB p65-driven inflammation and ferroptosis, indicating its potential therapeutic application in OA patients.

**Supplementary Information:**

The online version contains supplementary material available at 10.1186/s13020-025-01166-2.

## Background

Osteoarthritis (OA), the prevailing type of arthritis, impacts more than 500 million individuals globally and results in compromised quality of life and substantial economic implications [1, 2]. Clinically, OA is distinguished by degenerative cartilage and inflammation, leading to chronic physical disability, pain, and potential joint deterioration [3]. Prior to the advent of total joint replacement, there were no satisfactory disease-modifying drugs available for OA treatment [4, 5]. There are two approaches to resolve this issue. The first involves the development of innovative drugs that are designed based on the identification of a newly discovered marker gene specific to OA [6–8]. Although the development of novel drugs remains crucial for addressing OA, this approach is time-consuming and costly [9–11]. An alternative approach, which utilizes clinically established drugs, particularly those with anti-inflammatory properties for OA treatment, has garnered significant attention in recent years [12–14].

Sodium tanshinone IIA sulfonate (STS), a water-soluble compound, is derived from tanshinone IIA, which was found in the dried root of Salvia miltiorrhiza, a traditional Chinese medicine [15, 16]. The China State Food and Drug Administration (CFDA) has granted approval for the use of STS injection in the treatment of cardiovascular diseases in Chinese clinics for over three decades [17]. In addition to its established cardio-protective effects, STS exhibits various pharmacological properties, such as anti-inflammatory [18], anti-oxidative [19], endothelial protective [20], myocardial protective [21], anticoagulation [22], vasodilation [23], and anti-atherosclerosis [24] activities. STS has been found to inhibit inflammation and oxidative stress in ageing and degenerative diseases, such as Alzheimer’s disease [25]. Recently, STS has drawn more attention by its effects of inhibiting the proliferation, migration, invasion, and inflammation of fibroblast-like synoviocytes, thereby providing protection against rheumatoid arthritis [26, 27]. Despite the notable pharmacological effects and molecular mechanisms of STS exist in treatment of various diseases both in vivo and in vitro, its specific pharmacological treatments and mechanisms for OA treatment remain largely unknown.

Aging is widely recognized as the most prominent risk factor associated with OA development. SIRT1 is acknowledged as an anti-aging factor capable of extending the lifespan of mammals. It is extensively expressed in the superficial and middle zones of articular cartilage, and its protein level and activity decline as OA progresses [28]. In order to safeguard mice from OA, SIRT1 promotes cartilage anabolism while inhibiting cartilage catabolism and apoptosis [29]. Our research, as well as the research of others, has provided evidence for the significant and protective roles of SIRT1 in the development and maintenance of cartilage [30–33]. Thus far, SIRT1 has emerged as a potential target for drug therapy in OA treatment. Recent findings have indicated that STS may facilitate the upregulation of SIRT1 expression in human umbilical vascular endothelial cells and alveolar epithelial cells [20, 34]. However, the potential of STS to enhance SIRT1 expression in chondrocytes remains uncertain.

Aging-related diseases commonly exhibit an inflammatory pathogenesis, often referred to as "inflamm-aging", which may contribute to OA development, although the specific underlying mechanisms remain unidentified [35, 36]. Nuclear Factor Kappa B (NF-κB), a crucial intracellular nuclear transcription factor, plays a pivotal role in the regulation of cell survival genes and the coordination of inflammatory cytokine expression [37]. Recently, NF-κB has also been found to trigger ferroptosis, a distinct form of cell death characterized by the buildup of iron-dependent lipid peroxidation [38, 39]. It is known that SIRT1 deacetylates Nuclear Factor Kappa B Subunit p65 (NF-κB p65) at Lys310, thereby inhibiting its transcriptional activity. Although STS demonstrates the ability to mitigate inflammation through the upregulation of circ-*Sirt1* and the inhibition of NF-κB p65 translocation into the nucleus in LPS-treated RAW264.7 cells [40], the precise mechanism that STS uses to treat the SIRT1/NF-κB p65/inflammation/ferroptosis pathway in chondrocytes remains unclear.

The current study aimed to explore whether and how STS plays pharmacological roles in OA. We found that STS could delay articular cartilage degeneration, thereby attenuating OA progression. Mechanistically, STS increased SIRT1 protein expression and promoted SIRT1-mediated deacetylation of NF-κB p65, which then inhibited inflammation and ferroptosis in chondrocyte. This research sheds new light on the  therapeutic effects of STS on OA and its underlying molecular mechanisms.

## Materials and methods

### Primary chondrocyte culture and induction

The knee joint cartilage of newborn mice aged 1-3 days was dissected, rinsed in PBS, and subsequently digested in 0.2% collagenase (Sigma, C6885) at 37 °C for 1.5 h. The resulting cell suspension was repeatedly aspirated and filtered through a 70-μm cell strainer, followed by rinsing in PBS and serum-free Dulbecco’s modified Eagle's medium (DMEM) (Gibco, 11965-092). The cells were then counted. Chondrocytes were seeded at a density of 2 × 10^6^ cells/mL in DMEM supplemented with 100 units/mL penicillin, 100 μg/mL streptomycin (Beyotime, C0222), 10% fetal bovine serum (FBS) (OPCEL, BS-1102), and 2 mM glutamine (Solarbio, G0200). The culture medium was replaced every 48 h. Chondrocytes from passages 0 to 2 were utilized for analysis in this study. When the chondrocytes reached 80% confluence, ITS (Sigma, I3146) at a dilution of 1:100 was added to the cell culture medium for a duration of 7 days to stimulate chondrocyte differentiation and hypertrophy. The culture medium was refreshed every alternate day. To induce ferroptosis, chondrocytes were incubated with 5 μM Erastin (Beyotime, SC0224) for 24 h.

### Interleukin-1β (IL-1β) and STS treatment on chondrocytes

Following a 7-day treatment with ITS, the primary chondrocytes were treated with or without 100 μM of STS in the presence of IL-1β (10 ng/ml) for 24 h. Then the chondrocytes were collected for further analysis.

### Cell viability detection by MTT

For MTT assay, the procedures were conducted according to the MTT cell proliferation and Cytotoxicity Assay Kit (Solarbio, M1020). Primary chondrocytes were seeded into 96-well plates at a density of 1 × 10^4^ chondrocytes per well. After treatment with different concentrations of STS for 24 h, 10 μL MTT was added into each well to reach a working concentration at 0.5 mg/ml, and chondrocytes were incubated for additional 4 h. Finally, after the culture medium was removed, 110 μL Formazan liquid solution was added into each well, and optical density was measured at wavelength of 490 nm.

### RNA isolation and real-time quantitative PCR (RT-qPCR)

RNA was extracted by the Trizol method. For primary chondrocytes, after aspirating the medium, added 200 μL chloroform per 1 ml of Trizol Reagent (Invitrogen, 15596026CN) and centrifuged for 15 min at 4 °C, 15,700 g. The cartilage was rapidly frozen in liquid nitrogen prior to being subjected to treatment with the Trizol reagent mixture. Subsequently, the upper layer was transferred to a new tube and thoroughly mixed with an equal volume of isopropanol. After centrifugation for 25 min at a top speed, the supernatant was removed and 70% EtOH was added for RNA preparation. At last, the RNA quality was confirmed by using Nano-drop to ensure that the OD280/260 should be higher than 1.8. The reverse transcription process was performed utilizing the Revert Aid First Strand cDNA Synthesis kit (Thermo Fisher Scientific, K1621) in accordance with the manufacturer's guidelines. Subsequently, RT-qPCR was conducted using SYBR Premix Ex Taq II (Takara Bio, RR047A) as per the provided instructions. The assessment of target gene expressions was carried out employing a relative quantification approach, 2^–△△ct^ method, in comparison to the expression of β-actin. The primer sequences of the PCR reaction utilized in this study are detailed in Supplementary Table 1.

### Caspase 3 activity assay

The analysis on apoptosis of chondrocytes was conducted according to the protocols in the Caspase 3 Activity Assay Kit (Beyotime, C1115). Briefly, the chondrocytes were digested in 0.2% collagenase and centrifuged at 600 g 4 °C for 5 min. The precipitated chondrocytes were resuspended in cell lysate for 15 min on ice. After centrifugation at 16,000 g for 15 min, the enzyme activity of Caspase 3 was determined.

### *Sirt1* knockdown, inhibition, and activation in primary chondrocytes

To induce *Sirt1* knockdown, primary chondrocytes were incubated with 4-OH tamoxifen (TM) (Sigma, T176) (1 μM) for 1 week. Afterwards, 4-OH TM-incubated primary chondrocytes isolated from Col2a1-CreERT; *Sirt1*^*flox/flox*^ mice were designated as *Sirt1*KO chondrocytes. For SIRT1 activity inhibition or activation, primary chondrocytes were treated with 10 μM EX-527 (Selleck, S1541) or 10 μM SRT2104 (Selleck, S7792) for 24 h.

### Immunoprecipitation (IP) and western blotting

The nuclei lysate was obtained using a Nuclear and Cytoplasmic Protein Extraction Kit (Beyotime, P0027) following established protocols. Subsequently, the extracted nuclei lysate was incubated with the NF-κB p65 (acetyl Lys310) antibody (Abcam, ab19870) at a dilution of 1:500, coupled with Protein A/G PLUS-Agarose (Santa Cruz, sc-2003), as per the manufacturer’s instructions. Protein lysates from primary chondrocytes were extracted using RIPA lysis buffer (Beyotime, P0013C) and quantified using the Quick StartTM Bradford Protein Assay (BIO-RAD, 5000207). The extracted protein lysates were loaded and migrated on SDS-polyacrylamide (SDS-PAGE) gel (ACE, ET15412Gel) in running buffer (ACE, F00001GelS) at 160 V for 30 min. The proteins that had been separated were subsequently transferred onto a polyvinylidene difluoride (PVDF) membrane (Millipore, Billerica, MA, USA) using an electrical potential of 100 V for a duration of 0.5–2.5 h on ice. The PVDF membrane was then subjected to blocking with 5% milk and subsequently incubated with the designated  primary antibodies overnight at a temperature of 4 °C: β-actin (Proteintech, 66009-1-lg) (1:50,000), SIRT1 (Abcam, ab189494) (1:1000), NF-κB p65(CST, 8242) (1:10,000), NF-κB p65 (acetyl Lys310) (Abcam, ab19870) (1:10,000), Type II Collagen Alpha 1 Chain (COL2A1) (Proteintech, 28459-1-AP) (1:1000), Ferritin heavy chain 1 (FTH1) (Abcam, ab183781) (1:1000), Glutathione peroxidase 4 (GPX4) (Abcam, ab125066) (1:1000), acyl-CoA synthetase long-chain family member 4 (ACSL4) (Proteintech, 22401-1-AP) (1:5000), P300 (Santa Cruz, sc-48343)(1:1000). The PVDF membranes were subjected to three rounds of incubation with 1 × TBST for 10 min each. Subsequently, the membranes were incubated with the following secondary antibodies at room temperature for 1 h: Goat anti-rabbit IgG (ZHUANGZHI, EK020) (1:10,000) and Goat anti-mouse IgG (ZHUANGZHI, EK010) (1:10,000). The blots were ultimately visualized utilizing the ECL detection system (Kermey, M0102). A representative blot for each protein was selected from three or six independent experiments. Densitometry analysis was conducted using Image J software.

### Enzyme-linked immunosorbent assay (ELISA)

Serum was collected at the indicated time-points after intra-articular injection of STS at different concentrations. Chondrocytes and supernatants were harvested after the treatments. The levels of SIRT1, inflammatory cytokines, and ferroptosis-related markers were assessed using ELISA assay kits in accordance with the manufacturer's instructions. The ELISA kits included SIRT1 (MEIMAIN, MM-44881M1), IL-6 (MEIMAIN, MM-0163M1), IL-17A (MEIMAIN, MM-0759M1), TNF-α (MEIMAIN, MM-0132M1), Lipid peroxide (LPO) (Shanghai Yuanxin Biotech Co., Ltd., YX-13615M), Malondialdehyde (MDA) (Shanghai Yuanxin Biotech Co., Ltd., YX-130401M), Glutathione (GSH) (Shanghai Yuanxin Biotech Co., Ltd., YX-071908M), 4-Hydroxy-2-nonenal (4-HNE) ELISA Kit instruction (Shanghai Yaji Biotechnology Co., Ltd., YS03024B), and GPX4 Activity Assay Kit (Elabscience, E-BC-K883-M).

### Animal care and studies

The mice were housed in a controlled environment of an SPF-grade experimental animal room, with temperature (22 °C), humidity, and light (12 h of light/12 h of darkness) regulated, and were provided unrestricted access to both chow and water.

### Study approval

The animal experiments were carried out in accordance with the guidelines set by the Animal Experiment Administration Committee of Xi’an Jiaotong University in Shaanxi, People’s Republic of China (NO. 2021-1243).

### Destabilization of the medial meniscus (DMM) surgery

Male C57BL/6 mice were subjected to intraperitoneal administration of pentobarbital sodium for anesthesia, followed by shaving of the hair surrounding the joint prior to surgery. Then, the related procedures were conducted under a microscope. The knee was bent to 90° and the joint capsule was opened with a pair of micro-surgical scissors by making an incision along the medial patellar tendon with a #11 blade. Subsequently, the medial meniscus ligament was incised using a micro-surgical scalpel, resulting in the destabilization of the medial meniscus, commonly referred to as DMM. In the sham group, the mice were subjected to knee joint capsule exposure, which was subsequently closed using a 3/8 arc suture needle. One week later after the above procedure, the joint capsule of the mice in both groups was intra-articular injected with a single dose of STS (Nuo Xin Kang, H31022558) once a week for 8 weeks at different concentrations of 15 μg, 30 μg, and 45 μg.

### Walking test

The mice in each group were adopted to the open field for 30 min before the test. Each mouse was positioned within the center of individual plastic square chambers (100 cm × 100 cm) and allowed for exploring the chamber freely in 6 min. Their movements were real-time recorded by a video camera and the distances each mouse moving in 6 min were analyzed by the software Smart 3.0.

### Plantar test

The plantar test apparatus (Ugo Basile Inc.) was utilized to measure the paw-withdrawal latency in response to radiant heat. Prior to testing, mice were acclimated to the environment within a glass box for a duration of 1 h. The mean paw-withdrawal latencies of the hind paws were determined by calculating the average from three independent experiments, with intervals of 5–8 min between each experiment to avoid thermal sensitization. A cut-off time of 20 s was implemented to prevent any potential tissue damage.

### Quantitative histology

Following euthanasia, the knee joints of the mice in each experimental group were fixed in a 4% paraformaldehyde solution overnight. Subsequently, decalcification was carried out using a 10% EDTA solution for a period of 14 days at room temperature. The decalcified knee joints were then embedded in paraffin. To assess the condition of the cartilage in the medial tibial plateau, three sections of the knees were cut along the sagittal plane, with each section measuring 4 μm in thickness. These sections were stained using Safranin-O (Sigma, CAS 477-73-6) and Fast Green (Sigma, F7252) in a combined staining technique known as Safranin O–Fast Green. These sections were also subjected to hematoxylin–eosin (HE) staining using a HE staining kit (Beyotime, C0105S). The evaluation of the cartilage in the medial tibial plateau was performed using the Osteoarthritis Research Society International (OARSI) cartilage OA histopathology grading system. Three sections were selected from each knee for analysis.

### TUNEL detection

Apoptotic chondrocytes within the tibial articular cartilage and primary chondrocytes were identified using an in-situ cell death detection kit (Roche, 1168479590; Roche, 12156792910), following the manufacturer’s instructions. The sections were subjected to dewaxing and rehydration by heating at 60 °C, followed by xylene washing and rehydration through a series of graded ethanol solutions and double-distilled water. Prior to the Tunel Mix incubation, the tissues were treated with proteinase K for 30 min at 37 °C. The primary chondrocytes were fixed with paraformaldehyde for 1 h at 4 °C. The membrane was permeabilized using a 0.1% TritonX-100 solution for a duration of 2 min on ice, followed by incubation with Tunel Mix for 1 h at a temperature of 37 °C. Nuclei were counterstained using 4´, 6-diamidino-2-phenylindole. Chondrocytes that exhibited TUNEL-positive staining were visualized through red or green fluorescence. The percentage of TUNEL-positive chondrocytes relative to the total chondrocyte population was determined by calculating the average across three distinct grid locations. All measurements were conducted by an observer who was unaware of the treatment category.

### Generation of mice with targeted disruption of *Sirt1* specifically in cartilage

The mice used in this study were obtained from various sources. C57BL/6 mice were purchased from the animal laboratory of Xi'an Jiaotong University. *Sirt1*^*flox/flox*^ mice were kindly provided by Prof. Gao Xiang of Nanjing University. FVB-Tg (Col2a1-cre/ERT) KA3Smac/J mice (stock no. 006774) were purchased from The Jackson Laboratory and were backcrossed with C57BL/6 mice for at least 8 generations. To generate the experimental mice, *Sirt1*^*flox/flox*^ mice were bred with Col2a1-cre/ERT mice, resulting in the production of Col2a1-CreERT; *Sirt1*^*flox/flox*^ mice. The Col2a1-CreERT; *Sirt1*^*flox/flox*^ mice were viable and fertile after TM induction. To induce *Sirt1* knockout, TM (Sigma, T5648, 75 mg/kg) was intraperitoneally injected for five consecutive days into 8-week-old mice until dissection at 16 weeks of age. The *Sirt1*^*flox/flox*^ and Col2a1-CreERT; *Sirt1*^*flox/flox*^ mice received the identical amounts of TM to ensure consistency. Afterwards, the *Sirt1*^*flox/flox*^ mice were defined as control mice, and Col2a1-CreERT; *Sirt1*^−/−^ mice were defined as *Sirt1*cKO mice.

### Mice genotyping

The genomic DNA from mouse tail snips was extracted using the Mouse Direct PCR Kit (Bimake.com, B40015). Additionally, the genomic DNA from various tissues, such as the heart, liver, spleen, lung, kidney, and cartilage, was extracted using the DNeasy blood and tissue kit (Qiagen, 9504). Genotyping was performed by PCR through using the respective primers (Supplementary Table 2). In mutant mice, a 750-bp fragment of the *Sirt1* gene was detected using primers OIMR7909 and OIMR7912. A 900-bp fragment was found when the *Sirt1* gene was intact, while a 450-bp fragment was observed when it was deleted, using primers OIMR7911 and OIMR7912. Additionally, a 358-bp *Col2a1-CreER*^*T*^ gene fragment was detected in the mutant mice.

### Immunohistochemical assay

All the procedures were conducted according to the manufacturer’s protocols of SPlink Detection Kits (Zhongshan Golden Bridge Biotechnology, SP-9000). Briefly, knee sections were dewaxed and gradient hydrated, and then treated with 0.25% trypsin (Beyotime, C0205) for 15 min at 37 °C. Subsequently, the sections were incubated with H_2_O_2_ and subsequently blocked with 5% goat serum for a duration of 15 min at a temperature of 37 °C. Following this, the sections were subjected to overnight incubation with primary antibodies at a temperature of 4 °C. The primary antibody information is as follows: collagen type X alpha 1 (COL10A1) (Abcam, ab260040), matrix metalloproteinase-13 (MMP13) (Proteintech, 18165-1-AP), SIRT1 (FineTest, FNab07877), NF-κB p65 (acetyl Lys310) (GeneTex, GTX86963), ACSL4 (Proteintech, 22401-1-AP). The primary antibodies were diluted by using Diluent Solution (Beyotime, P0262). On the subsequent day, following three rinses with phosphate-buffered saline (PBS), the sections were incubated with a working solution of secondary antibody for a duration of 15 min at a temperature of 37 °C. For visualization, diaminobenzidine (Zhongshan Golden Bridge Biotechnology, AL1-9018) was added to the slides. Finally, the nuclei of the sections were counterstained using hematoxylin, and the results were observed under a microscope (Leica, DFC310 FX). The entire cartilage tissue of each slice was observed under 100 × magnification. Two fields of vision were selected under 400-fold magnification for each slice and were captured using a digital slice scanning system (3DHISTECH, CaseViewer 2.4). The positive expression DAB appeared as brown-yellow, while the nuclei were stained with hematoxylin. Data analysis was performed using a microscopy image analysis system (Indica labs, Halo 101-WL-HALO-1). To analyze the positive area of COL10A1 and MMP13, the percentage of positive staining in each image was calculated (% DAB Positive Tissue). To analyze the positive expression of SIRT1, NF-κB p65, and ACSL4 in cartilage, the positive cells were counted in each field. Average values from the two visual fields were calculated for each slice. All measurements were conducted by a single observer who was blinded to grouping.

### Fe^2+^ detection

The intracellular iron (Fe^2+^) levels of chondrocytes were measured using the iron assay kit (Abcam, ab83366) according to the manufacturer’s instructions. Chondrocytes were seeded in six-well plates at a density of 1 × 10^6^/well with various treatments. Then chondrocytes were harvested in cold PBS, resuspended in Iron assay buffer at a concentration of 1 × 10^6^ per 100 μL, and homogenized on ice. The resulting homogenate was subjected to centrifugation at 16,000 g for 10 min at 4 °C, and the resulting supernatant was used for the assay. Subsequently, 100 μL of supernatant and 100μL of standard solution were added to a 96-well plate. Iron Reducer (5 μL) was added to each standard well, while Assay buffer (5μL) was added to each supernatant well. The mixture was thoroughly combined and incubated at 37 °C for 30 min. At last, the absorbance was measured using a microplate reader (BioTek, Epoch) at a wavelength of 593 nm.

### Reactive oxygen species (ROS) detection

The intracellular levels of ROS in chondrocytes were quantified using an ROS assay kit (Beyotime, S0033S) with the fluorescent probes DCFH-DA, following the manufacturer’s protocol. Chondrocytes with different treatments were initially seeded in 6-well plates at a density of 1 × 10^6^ cells per well. Subsequently, the cells were added with a 1 mL diluted solution of DCFH-DA probe (10 μM/L) and incubated at 37 °C for 20 min. The cells were then washed three times with serum-free medium, digested, resuspended in 100 μL PBS, and transferred to a 96-well black, clear bottom plate (Beyotime, FCP965) for analysis. The fluorescence intensity was measured at a microplate reader (Tecan, Spark) with excitation and emission wavelengths at 488 nm and 525 nm, respectively.

### Plasmid information and transfection

The NF-κB p65 and NF-κB p65 (K310R) plasmids were synthesized by Haixing Biosciences Co., Ltd. (Jiangsu, China) utilizing the NF-κB p65 gene (Gene ID: 19697). These plasmids were subsequently transfected into chondrocytes using Lipofectamine 3000 (Invitrogen, USA) in accordance with the manufacturer’s protocol.

### Transmission electron microscopy experiments

The collected chondrocytes were cut into 1 mm^3^ cubes, fixed in a glutaraldehyde and paraformaldehyde solution at 4 °C for 2 h, then washed and post-fixed in osmium tetroxide. After rinsing, they were dehydrated in increasing ethanol concentrations, stained, further dehydrated, replaced with propylene oxide, and finally embedded in epoxy resin Epon812 for polymerization. Semi-thin sections (1–2 μm) were stained with methylene blue and examined with an optical microscope. Ultra-thin sections (50–70 nm) were prepared using a Leica UC7 ultramicrotome, stained with uranyl acetate and lead citrate, and imaged with a HITACHI H-7650 transmission electron microscope.

### Statistical analysis

Statistical analysis and graphical representations were conducted using GraphPad Prism 9.0 software. The data were presented as means ± standard deviation (SD). The normality of the data was assessed using the D’Agostino & Pearson test, while the homogeneity of variance was evaluated using the F test. For comparisons between two groups, an unpaired two-tailed Student’s t-test was employed, whereas for comparisons involving more than two groups, a one-way analysis of variance (ANOVA) with Dunnett’s correction was utilized. A significance level of *P* < 0.05 was deemed statistically significant.

## Results

### STS alleviated IL-1β-induced matrix degradation and apoptosis in chondrocytes

**Fig. 1:**
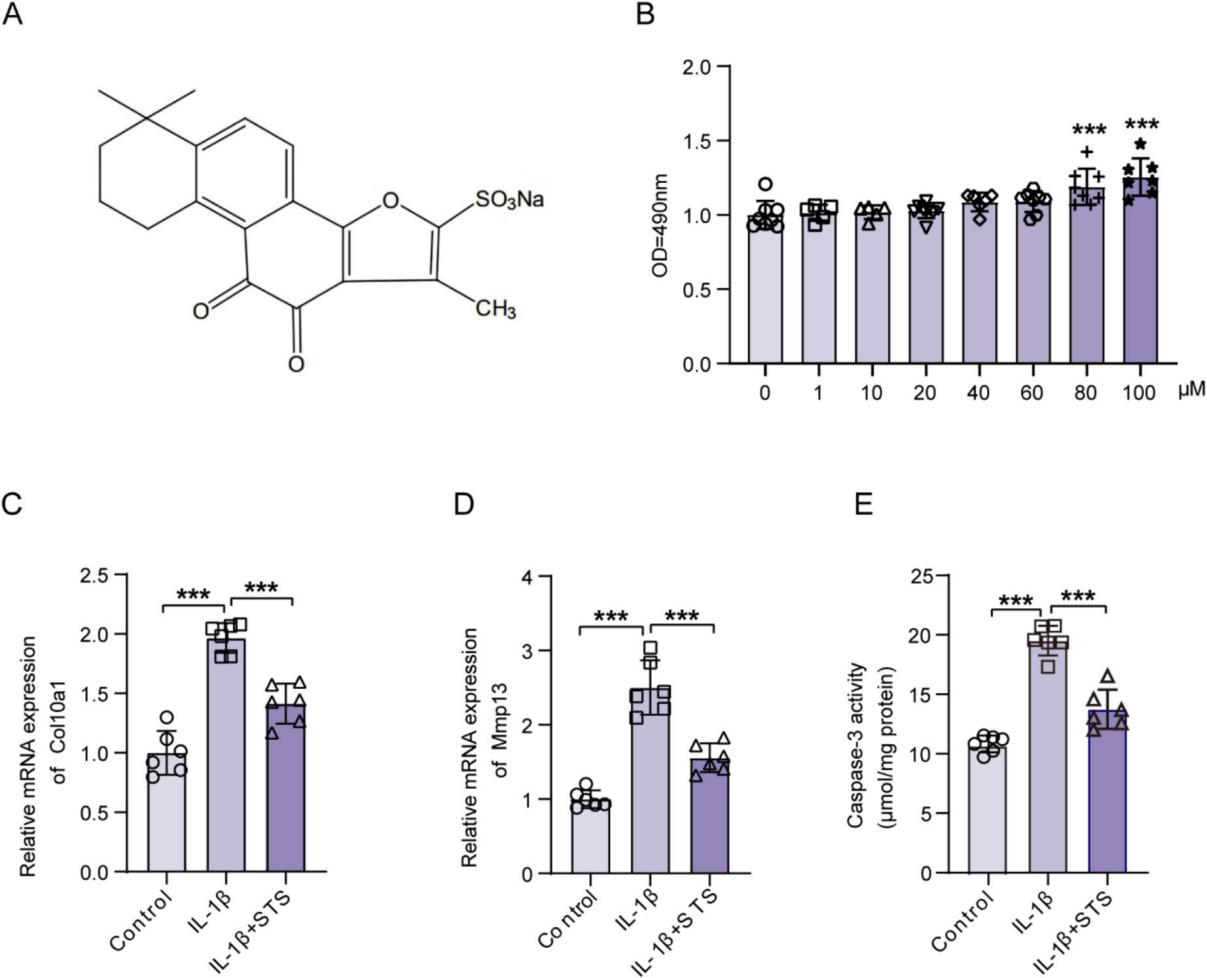
STS inhibited IL-1β-induced matrix degradation and apoptosis in primary chondrocytes. **A** The Molecular structure of STS. **B** Cell survival assay of primary chondrocytes treated with gradient concentrations of STS (n = 5–7/group) by MTT. **C, D** Relative mRNA expression of *Col10a1* and *Mmp13* from primary chondrocytes in each group (Control, IL-1β, IL-1β + STS) (n = 6/group). **E** Quantification of Caspase-3 activity from primary chondrocytes in each group (Control, IL-1β, IL-1β + STS) (n = 6/group). Data are means ± SD, One way-ANOVA; ***P < 0.001

To examine the effects of STS on OA, STS was used to treat primary chondrocytes (Fig. 1A). The effective concentrations of STS on chondrocytes were initially determined using an MTT assay (Fig. 1B). Additionally, our findings indicated that STS had no significant impact on chondrocyte function at 80 μM, but significantly stimulated COL2A1 (a chondrocyte marker) at 100 μM, as assessed by western blotting (Fig. S1). Based on these results, we selected a concentration of 100 μM for subsequent studies. To mimic OA pathological condition in vitro, chondrocytes were treated with IL-1β in the presence or absence of STS. The expression of COL10A1 and MMP-13, markers of cartilage hypertrophy and degradation, apparently increased in the primary chondrocytes induced by IL-1β, indicating chondrocyte matrix degradation (Fig. 1C-D), with these effects being nullified by the addition of STS (Fig. 1C-D). As apoptosis is one of the pathological manifestations of OA, we further investigated the effects of STS on the apoptotic chondrocytes. STS treatment significantly reduced apoptosis of the primary chondrocytes induced by IL-1β, as demonstrated by the Caspase-3 activity (Fig. 1E). These results indicate that STS can alleviate chondrocyte matrix degradation and apoptosis.

### STS promoted SIRT1 to alleviate OA progression

**Fig. 2:**
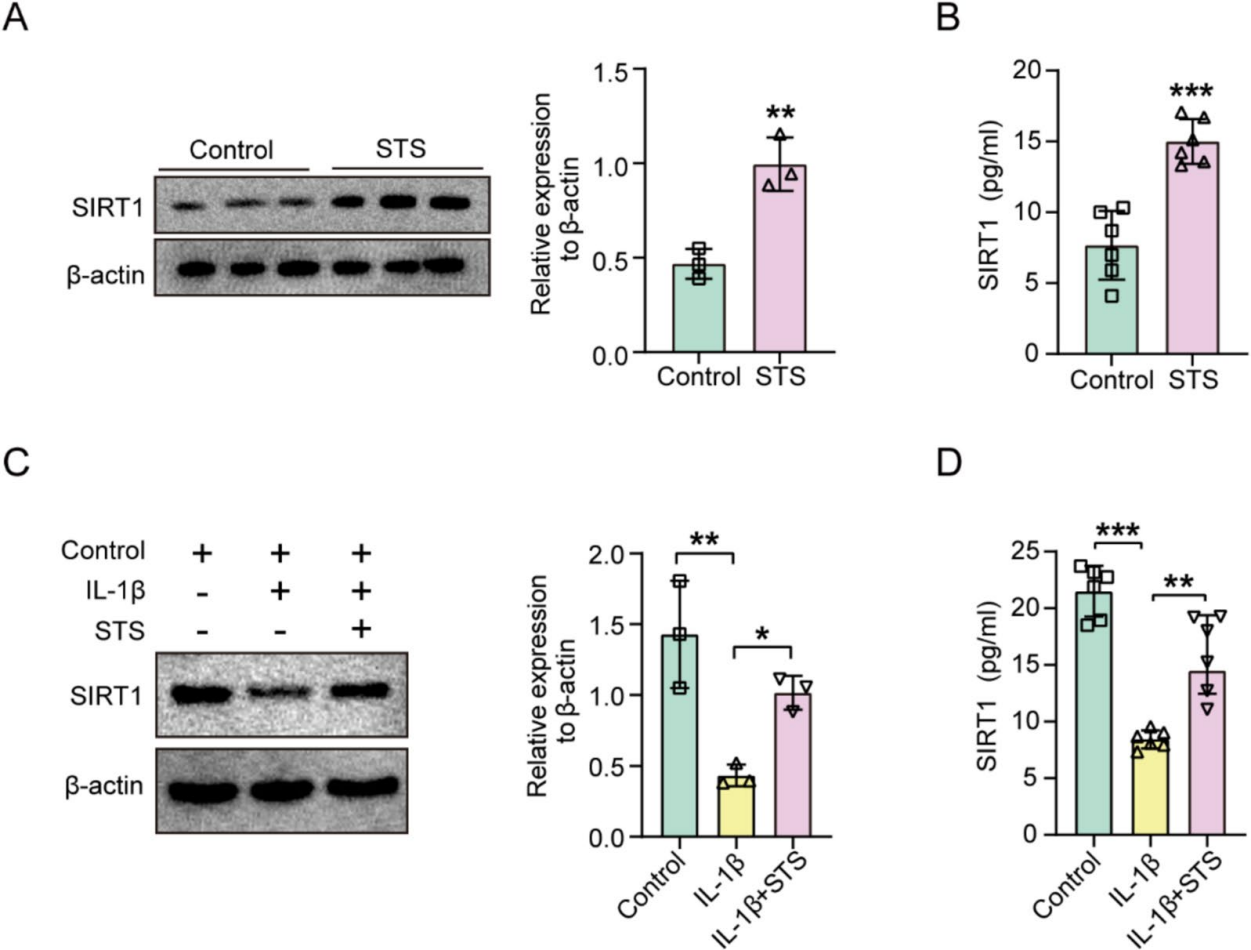
STS increased SIRT1 expression in primary chondrocytes. **A** Protein expression of SIRT1 in the primary chondrocytes upon STS treatment. Right: Quantification of SIRT1 protein expression in the primary chondrocytes upon STS treatment (n = 3/group). **B** Concentration of SIRT1 in the supernatant of primary chondrocytes upon STS treatment (n = 6/group). **C** Protein expression of SIRT1 in the IL-1β-induced primary chondrocytes upon STS treatment. Right: Quantification of SIRT1 protein expression in the IL-1β-induced primary chondrocytes upon STS treatment (n = 3/group). **D** Concentration of SIRT1 in the supernatant of IL-1β-induced primary chondrocytes upon STS treatment (n = 6/group). Data are means ± SD. Student’s t-test for two groups; one-wayANOVA for three groups; *P < 0.05, **P < 0.01, ***P < 0.001

As SIRT1 is vital in cartilage homeostasis and exerts chondroprotective functions in OA, we thus hypothesized that STS may increase SIRT1 expression in chondrocytes to alleviate OA. We further detected the SIRT1 expression under STS treatment in the primary chondrocytes. Results indicated that STS significantly increased the protein expression of SIRT1 in primary chondrocytes (Fig. 2A) and improved the concentration of SIRT1 in the supernatant (Fig. 2B).

We then examined the effects of STS on SIRT1 expression in the primary chondrocytes treated with IL-1β. Upon IL-1β treatment, the protein expression of SIRT1 was significantly decreased, while STS effectively elevated its protein expression (Fig. 2C). The concentration of SIRT1 in supernatant exhibited similar trends (Fig. 2D)*.*

These results indicate that STS plays a protective role in OA progression through promoting SIRT1.

### STS promoted SIRT1 deacetylation of NF-κB p65, thereby inhibiting NF-κB-driven inflammation and ferroptosis to alleviate OA progression

Given that SIRT1 has been shown to deacetylate NF-κB to confer protection against OA [28], we sought to determine whether STS facilitates the deacetylation of NF-κB p65 by SIRT1, thereby exerting a protective effect against OA.

**Fig. 3:**
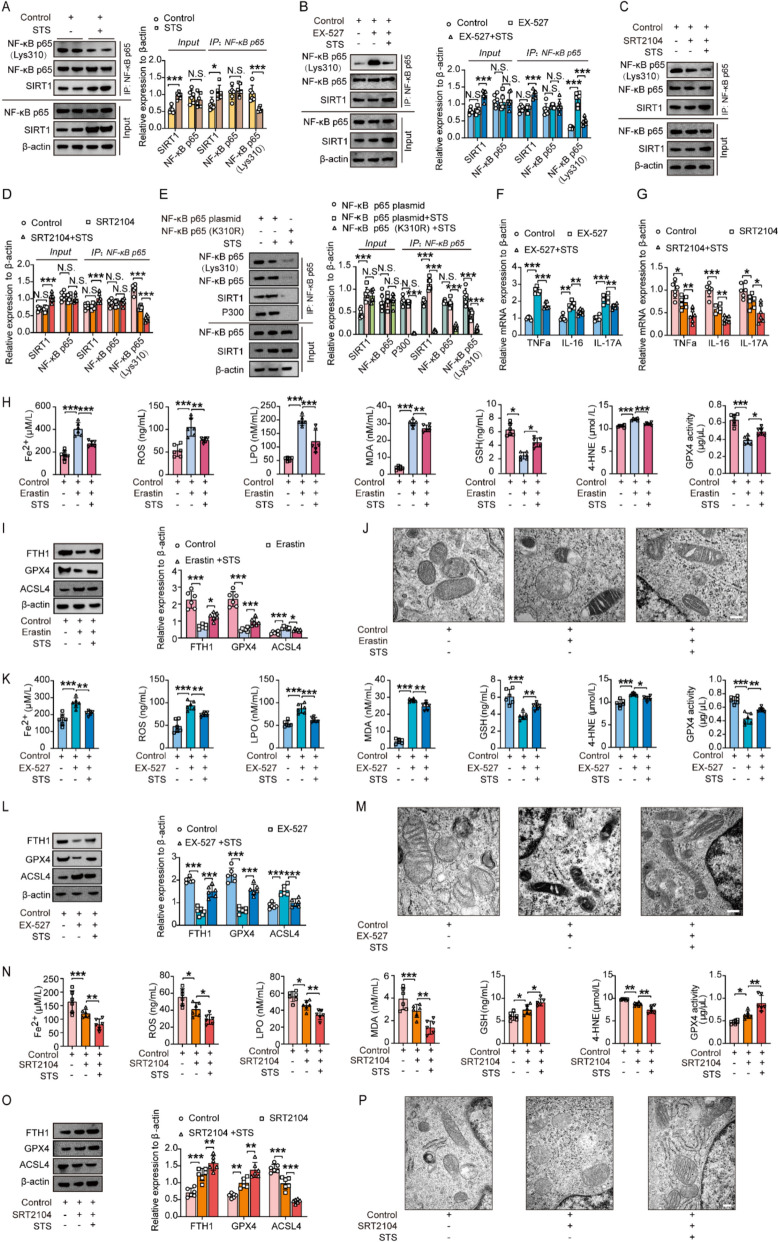
STS promoted SIRT1-mediated deacetylation of NF-κB p65, thereby inhibiting inflammation and ferroptosis in chondrocytes. **A** The nuclear protein levels of SIRT1, NF-κB p65, and acetylated NF-κB p65 (Lys310) immunoprecipitated from primary chondrocytes treated with or without STS. Right: Quantification of these proteins (n = 6/group). **B** The nuclear protein levels of SIRT1, NF-κB p65, and acetylated NF-κB p65 (Lys310) immunoprecipitated from primary chondrocytes treated with EX-527 and/or STS. Right: Quantification of these proteins (n = 6/group). **C** The nuclear protein levels of acetylated NF-κB p65 (Lys310) immunoprecipitated from primary chondrocytes treated with SRT2104 and/or STS. **D** Quantification of these proteins in group C (n = 6/group). **E** The protein levels of P300, SIRT1, NF-κB p65, and acetylated NF-κB p65 (Lys310) immunoprecipitated from primary chondrocytes treated with NF-κB p65 plasmid/ NF-κB p65 (K310R) plasmid and/or STS. Right: Quantification of these proteins (n = 6/group). **F** The relative mRNA expression of TNF-α, IL-6, and IL-17A in primary chondrocytes treated with EX-527 and/or STS (n = 6/group). **G** The relative mRNA expression of TNF-α, IL-6, and IL-17A in primary chondrocytes treated with SRT2104 and/or STS (n = 6/group). **H** The concentrations of Fe^2+^, ROS, LPO, MDA, GSH, 4-HNE, and GPX4 in the primary chondrocytes treated with Erastin and/or STS (n = 6/group). **I** The protein level of FTH1, GPX4, and ACSL4 from primary chondrocytes treated with Erastin and/or STS. Right: Quantification of these proteins (n = 6/group). **J** Representative images of mitochondrial morphology of the primary chondrocytes treated with Erastin and/or STS. Scale bar, 200 μm. **K** The concentrations of Fe^2+^, ROS, LPO, MDA, GSH, 4-HNE, and GPX4 in the primary chondrocytes treated with EX-527 and/or STS (n = 6/group). **L** The protein level of FTH1, GPX4, and ACSL4 from primary chondrocytes treated with EX-527 and/or STS. Right: Quantification of these proteins (n = 6/group). **M** Representative images of mitochondrial morphology of the primary chondrocytes treated with EX-527 and/or STS. Scale bar, 200 μm. **N** The concentrations of Fe^2+^, ROS, LPO, MDA, GSH, 4-HNE, and GPX4 in the primary chondrocytes treated with SRT2104 and/or STS (n = 6/group). **O** The protein levels of FTH1, GPX4, and ACSL4 from primary chondrocytes treated with SRT2104 and/or STS. Right: Quantification of these proteins (n = 6/group). **P** Representative images of mitochondrial morphology from the primary chondrocytes treated with SRT2104 and/or STS. Scale bar, 200 μm. Data are means ± SD, one-wayANOVA for three groups; N.S., no significant differences, *P < 0.05, **P < 0.01, ***P < 0.001

After STS treatment, SIRT1 expression increased, but NF-κB p65 levels remained unchanged. IP analysis showed SIRT1 directly interacts with NF-κB p65, significantly reducing NF-κB p65 (Lys310) acetylation, suggesting STS promotes SIRT1-mediated deacetylation of NF-κB (Fig. 3A). Blocking SIRT1 with EX-527 did not alter SIRT1 or NF-κB p65 levels, but increased acetylated NF-κB p65 (Lys310), with these effects being reversed by STS (Fig. 3B). Conversely, activating SIRT1 with SRT2104 enhanced NF-κB p65 (Lys310) deacetylation in chondrocytes, an effect amplified by STS (Fig. 3C, D). To examine the potential impact of STS on the deacetylation of SIRT1 at the NF-κB p65 site, we utilized site-directed mutagenesis. Specifically, we generated a mutant NF-κB p65 in which the lysine (K) residue at position 310 was substituted with arginine (R). The wild-type NF-κB p65 plasmid or K310R mutant plasmid was transfected into chondrocytes that had been treated with STS (Fig. 3E). Given that p300 is known to acetylate NF-κB p65 at K310, our findings indicate that the K310R mutation impairs the acetylation of NF-κB p65 by p300 at this site (Fig. 3E). Consequently, in the absence of acetylation at this site, SIRT1 deacetylation could not occur (Fig. 3E). Our results, therefore, indicate that the K310R mutation disrupts the regulatory effects of STS on SIRT1-mediated deacetylation of NF-κB p65 at K310 (Fig. 3E). These results suggest that STS effectively enhances the deacetylation of SIRT1 on NF-κB p65 at the K310 site.

Recent studies show that SIRT1 deacetylates NF-κB p65, reducing its transcriptional activity and inflammation [23–25]. Inhibiting SIRT1 with EX-527 increases NF-κB p65 acetylation and inflammatory cytokines (TNF-α, IL-6, IL-17A), while STS reverses these effects (Fig. 3B, F). Activating SIRT1 with SRT2104 decreases both NF-κB p65 acetylation and cytokine expression, with STS further enhancing these reductions (Fig. 3C, D, G). These findings suggest that NF-κB p65 acetylation regulates inflammatory factors, highlighting STS's anti-inflammatory effects via SIRT1-mediated deacetylation.

Studies indicated that ferroptosis can trigger OA and NF-κB can induce ferroptosis [39, 41]. Erastin was used to induce ferroptosis and reveal that STS could inhibit ferroptosis in primary chondrocytes (Fig. 3H). This inhibition was evidenced by the changes in the expression levels of Fe^2+^, ROS, LPO, MDA, and 4-HNE (positive markers of ferroptosis), as well as GSH and GPX4 (negative markers of ferroptosis) (Fig. 3H) [20]. The protein levels of FTH1, GPX4, and ACSL4 further supported this finding (Fig. 3I). Additionally, Erastin induction resulted in altered mitochondrial morphology, characterized by increased mitochondrial membrane density, reduction or loss of mitochondrial cristae, and rupture of the outer mitochondrial membrane (Fig. 3J). However, STS treatment effectively mitigated these morphological changes (Fig. 3J). Subsequent analysis of ferroptosis levels in the presence of EX-527 and SRT2104, with or without STS, showed a consistent pattern with acetylated NF-κB p65, which was regulated by SIRT1 (Fig. 3B, C, D, K, L, M, N, O, P). Our findings suggest a positive correlation between acetylated NF-κB p65 levels and ferroptosis, with STS inhibiting ferroptosis by facilitating SIRT1-mediated deacetylation of NF-κB p65 at K310.

**Fig. 4:**
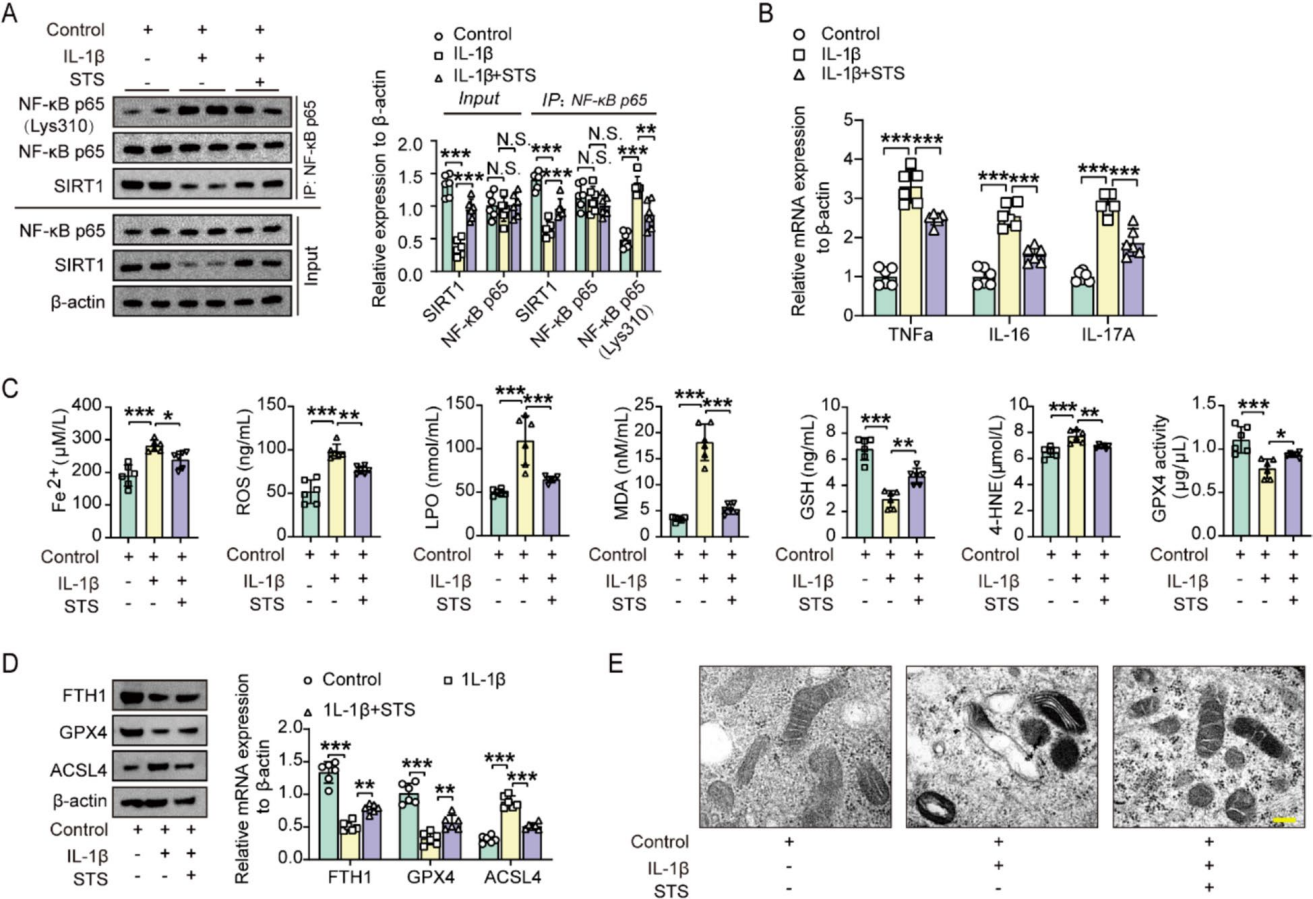
STS promoted SIRT1 deacetylation of NF-κB p65, thereby inhibiting inflammation and ferroptosis to alleviate OA. **A** Nuclear protein level of NF-κB p65 (Lys310) immunoprecipitated from primary chondrocytes treated with IL-1β and/or STS. Right: Quantification of the nuclear protein levels of the SIRT1, NF-κB p65, and acetylated NF-κB p65 (Lys310) immunoprecipitated from primary chondrocytes treated with IL-1β and/or STS (n = 6/group). **B** The relative mRNA expression of TNF-α, IL-6, and IL-17A in primary chondrocytes treated in the presence of IL-1β with or without STS (n = 6/group).** C** Concentrations of Fe^2+^, ROS, LPO, MDA, GSH, 4-HNE, and GPX4 in the primary chondrocytes of each group (Control, IL-1β, IL-1β + STS) (n = 6/group). **D** Protein levels of FTH1, GPX4, and ACSL4 from primary chondrocytes treated with 1L-1β and/or STS. Right: Quantification of these proteins (n = 6/group). **E** Representative images of mitochondrial morphology from the primary chondrocytes treated with 1L-1β and/or STS. Scale bar, 200 μm. Data are means ± SD. One-wayANOVA for three groups; N.S., no significant differences, *P < 0.05, **P < 0.01, ***P < 0.001

We then investigated the effects of STS on primary chondrocytes under pathophysiological conditions induced by IL-1β. IL-1β treatment reduced SIRT1 expression and increased acetylated NF-κB p65 (Lys310) without changing NF-κB p65 protein levels (Fig. 4A). STS reversed these effects, promoting SIRT1-mediated deacetylation on NF-κB p65 (Lys310) (Fig. 4A). Additionally, IL-1β increased mRNA levels of inflammatory cytokines TNF-α, IL-6, and IL-17A, which were significantly reduced by STS (Fig. 4B).

We also observed that STS reduced IL-1β-induced chondrocyte ferroptosis, as shown by changes in Fe^2+^, ROS, LPO, MDA, GSH, 4-HNE, GPX4, FTH1, and ACSL4 levels, as well as mitochondrial morphology (Fig. 4C-E).

Altogether, these findings suggest that STS promotes SIRT1-mediated deacetylation of NF-κB p65, subsequently inhibiting NF-κB-driven downstream inflammation and ferroptosis, thereby alleviating OA.

### STS alleviated OA progression in DMM-induced mice

**Fig. 5:**
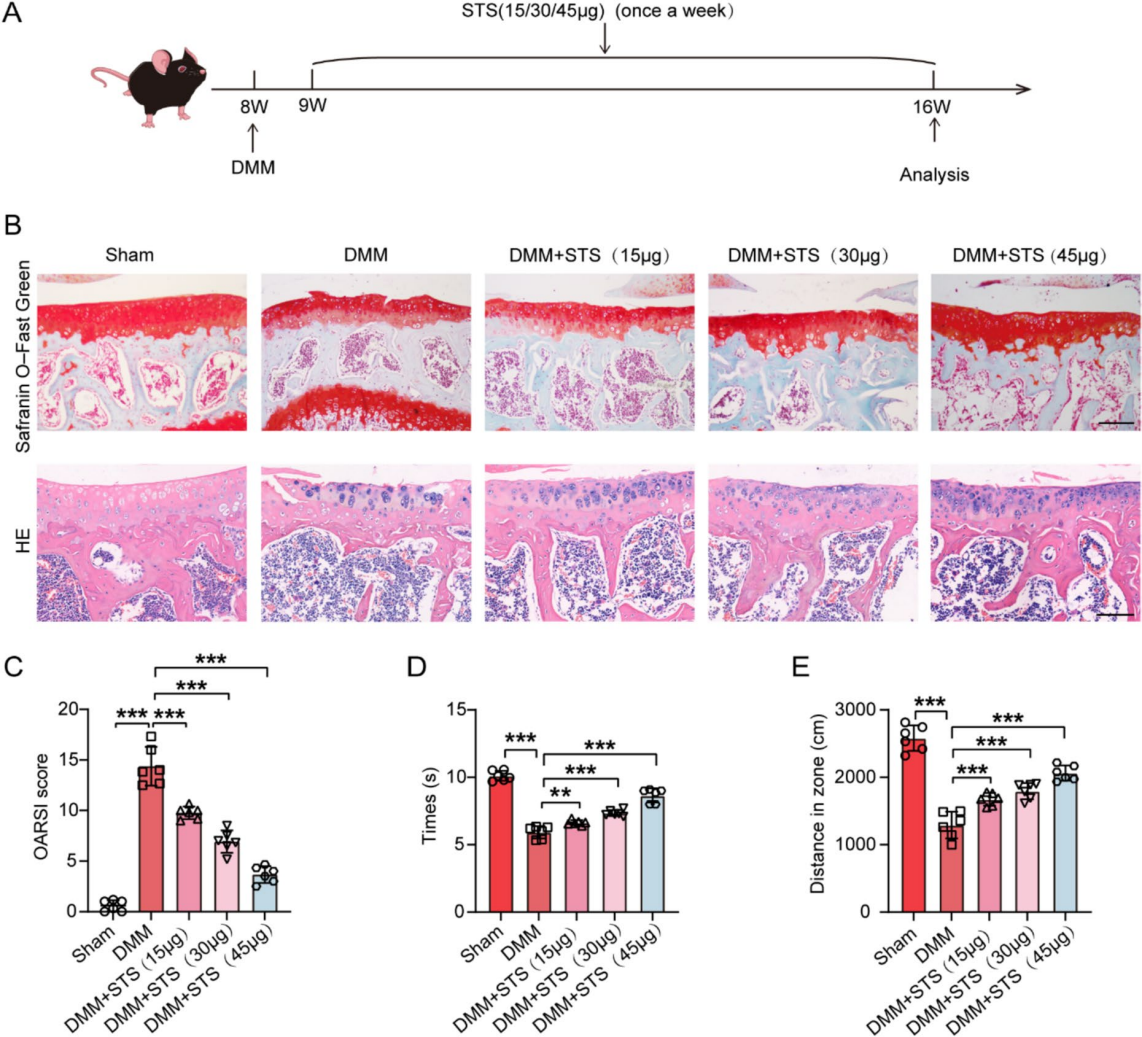
STS alleviated OA progression in DMM-induced mice. **A** Schematic illustration of animal experimental design. Schematic diagram of mice subjected to DMM surgery in the right knee, followed by treatment via intra-articular administration of STS (15/30/45 μg). **B** Representative images of Safranin O–Fast Green and HE staining of tibial articular cartilage in mice of each group. Bar = 100 μm. **C** Quantification of the OARSI score in each group (n = 6/group). **D** Plantar test of each group (n = 6/group). **E** Walking test of each group (n = 6/group). Data are means ± SD, One- wayANOVA; **P < 0.01, ***P < 0.001

To identify the effects of STS on OA progression in animal OA models, we intra-articularly injected STS into the knee joints of OA mice after DMM surgery at indicated time points (Fig. 5A). STS had no obvious side effects on mice, as no damage was observed in major organs (heart, liver, spleen, lung, and kidney) (Fig. S2). Tibial articular cartilage was moderately destroyed in DMM-induced mice, however, with these impairments being effectively attenuated by STS, as analyzed by Safranin O–Fast Green staining, HE staining, and OARSI grading (Fig. 5B, C). Pain and disability are hallmarks of OA, we thus assessed these two hallmarks in our mice. Plantar test and walking test indicated that STS effectively reduced the pain and the disability of the mice after DMM-surgery (Fig. 5D, E). These results demonstrate that STS effectively alleviated OA progression in a dose-dependent manner (from 15 to 45 μg). Thus, the STS at 45 μg for 8 weeks was used in the subsequent study.

### STS alleviated the cartilage degradation and apoptosis in OA mice

**Fig. 6:**
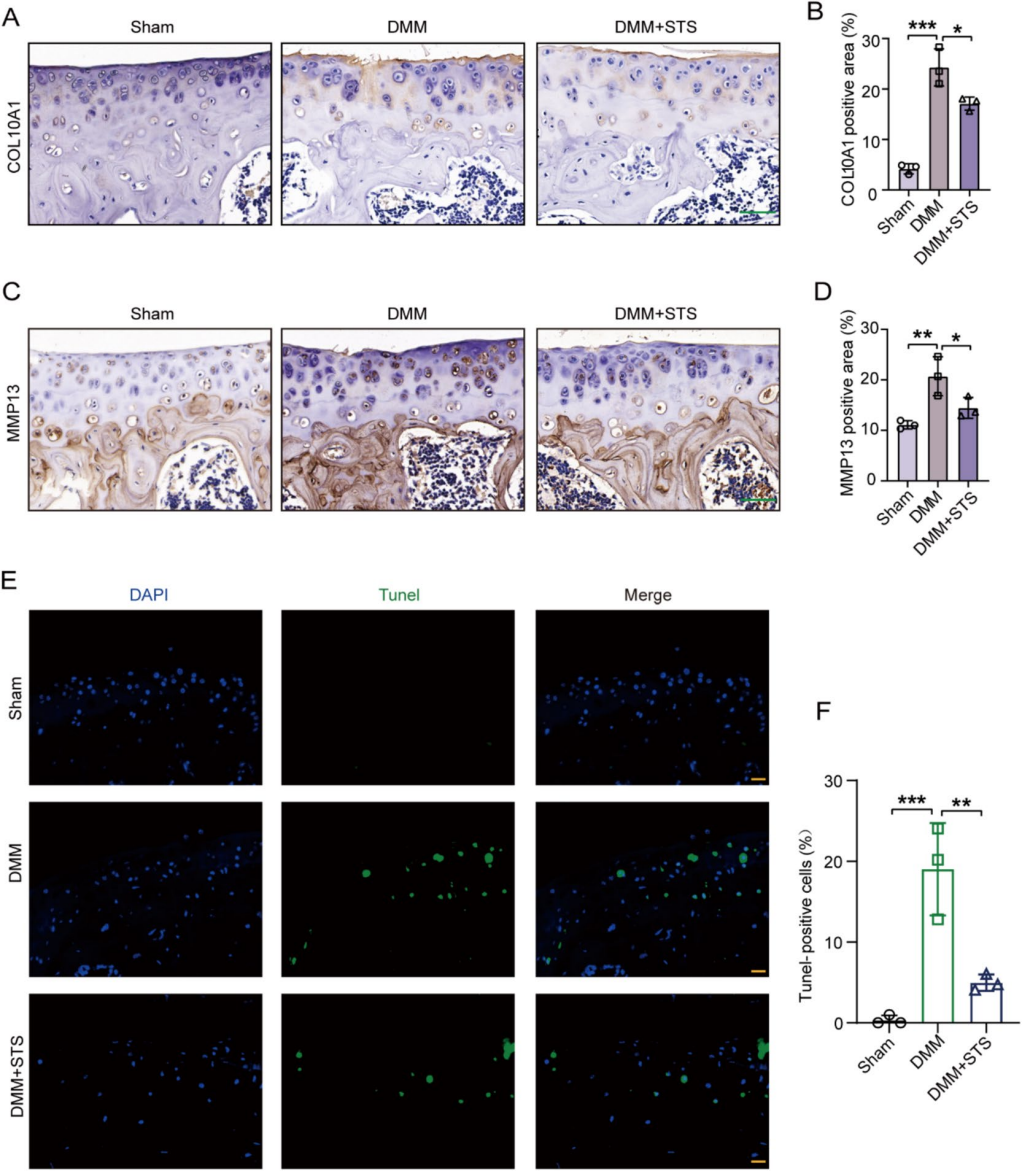
STS alleviated the cartilage degradation and apoptosis of OA in DMM-induced mice. An STS dose of 45 μg was used in the study. **A** Representative images of COL10A1 staining in tibial articular cartilage of mice for each group (Sham, DMM, DMM + STS). Bar = 50 μm. **B** Quantification of COL10A1 positive staining area in each group (Sham, DMM, DMM + STS) (n = 3/group). **C** Representative images of MMP13 staining in tibial articular cartilage of mice for each group (Sham, DMM, DMM + STS). Bar = 50 μm. **D** Quantification of MMP13 positive staining area in each group (Sham, DMM, DMM + STS) (n = 3/group). **E** Representative images of Tunel staining in tibial articular cartilage of each group (Sham, DMM, DMM + STS). Bar = 20 μm. **F** Quantification of Tunel positive staining chondrocytes in each group (Sham, DMM, DMM + STS) (n = 3/group). Data are means ± SD, One-wayANOVA; *P < 0.05, **P < 0.01, ***P < 0.001

To further examine the effects of STS on OA, we assessed the gene expression and the apoptosis in tibial articular cartilage under the treatment of STS. The expression of COL10A1 and MMP13 was apparently increased in the cartilage after DMM surgery, indicating cartilage matrix degradation (Fig. 6A–D), however, STS effectively normalized these protein levels of cartilage following DMM surgery (Fig. 6A–D). Meanwhile, STS significantly reduced the number and the percentage of the Tunel-positive apoptotic chondrocytes in the DMM model (Fig. 6E, F). These results indicate that STS can alleviate cartilage degradation and apoptosis to protect against OA.

### STS targeted SIRT1 / NF-κB p65/ inflammation/ferroptosis to alleviate OA progression

**Fig. 7:**
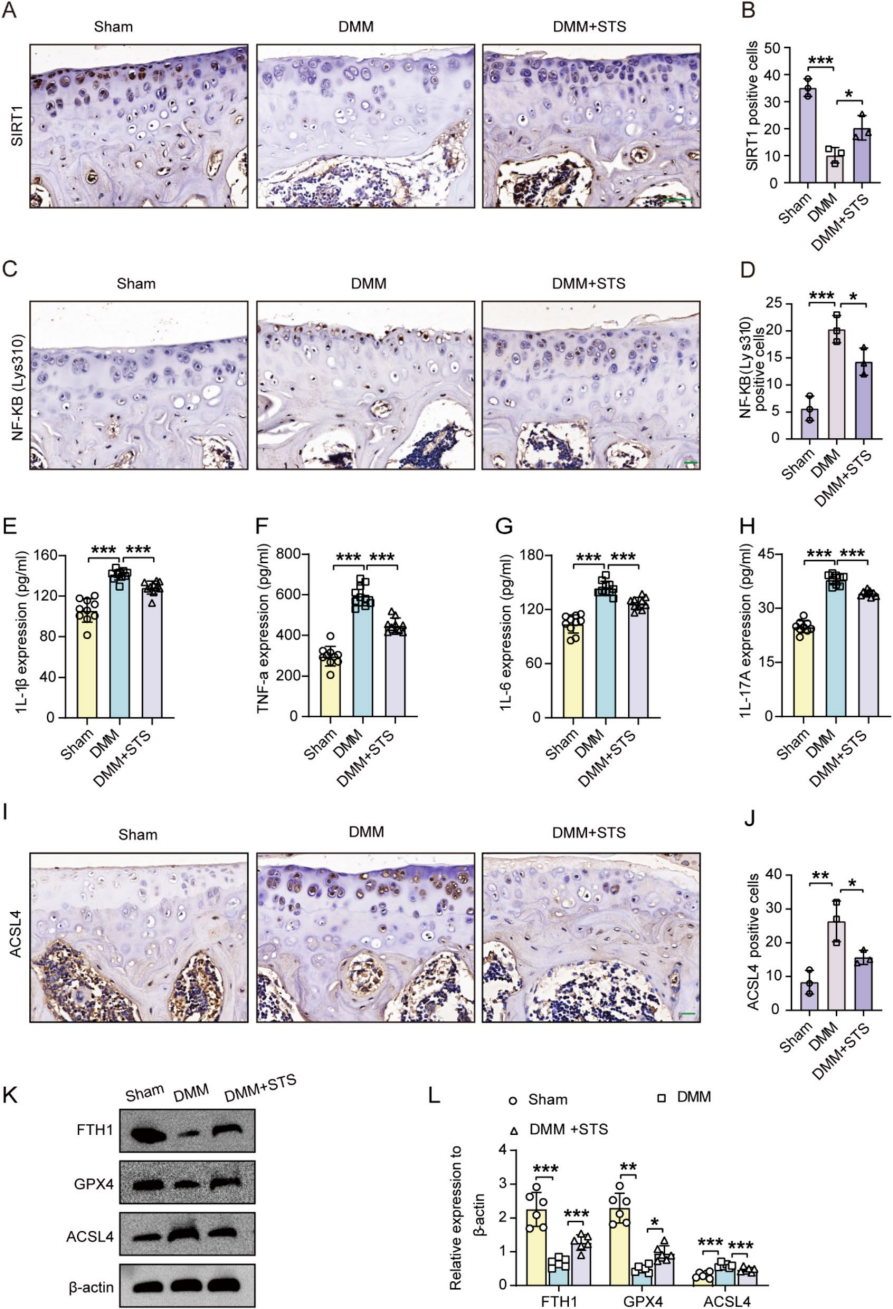
STS promoted SIRT1-mediated deacetylation of NF-κB p65, thereby inhibiting inflammation and ferroptosis to alleviate OA progression in DMM-induced mice. The STS of 45 μg was used in the study. **A** Representative images of SIRT1 staining in tibial articular cartilage of mice for each group (Sham, DMM, DMM + STS). Bar = 50 μm. **B** Quantification of SIRT1 positive chondrocytes in the tibial articular cartilage of mice for each group (Sham, DMM, DMM + STS) (n = 3/group). **C** Representative images of NF-κB p65 (Lys310) staining in tibial cartilage of DMM-induced mice treated with or without STS. Bar = 20 μm. **D** Quantification of NF-κB p65 (Lys310) positive chondrocytes in the mice tibial cartilage of each group (n = 3/group). **E–H** The serum expression of IL-1β, TNF-α, IL-6, and IL-17A in the mice of each group (Sham, DMM, DMM + STS) (n = 10/group). **I** Representative images of ACSL4 staining in the tibial articular cartilage of DMM-induced mice treated with or without STS. Bar = 20 μm. **J** Quantification of ACSL4 expression in the tibial articular cartilage of mice in each group (n = 3/group). **K** The protein levels of FTH1, GPX4, and ACSL4 from tibial articular cartilage of DMM-induced mice treated with or without STS. **L** Quantification of these proteins (n = 6/group). Data are means ± SD, One-wayANOVA; *P < 0.05, **P < 0.01, ***P < 0.001

Given SIRT1's role in cartilage homeostasis, we investigated whether STS could mitigate OA progression through SIRT1. Post-DMM surgery, SIRT1 expression in tibial cartilage decreased, but STS countered this reduction (Fig. 7A, B). Concurrently, NF-κB p65 (Lys310) levels increased during DMM surgery, with these effects being reversed by STS (Fig. 7C, D). STS treatment reduced elevated serum inflammatory cytokines (IL-1β, TNF-α, IL-6, IL-17A) observed in the DMM group compared to the Sham group (Fig. 7E-H). Additionally, DMM surgery triggered ferroptosis, but STS effectively reduced it, as shown by protein levels of ACSL4, FTH1, and GPX4 in tibial cartilage (Fig. 7I-L).

Consistent with the in vitro results, these findings indicate that STS can alleviate OA by increasing SIRT1 protein expression, promoting deacetylation on NF-κB p65 (Lys310), and inhibiting NF-κB-driven downstream inflammation and ferroptosis.

### STS failed to alleviate OA progression in *Sirt1*cKO mice

**Fig. 8:**
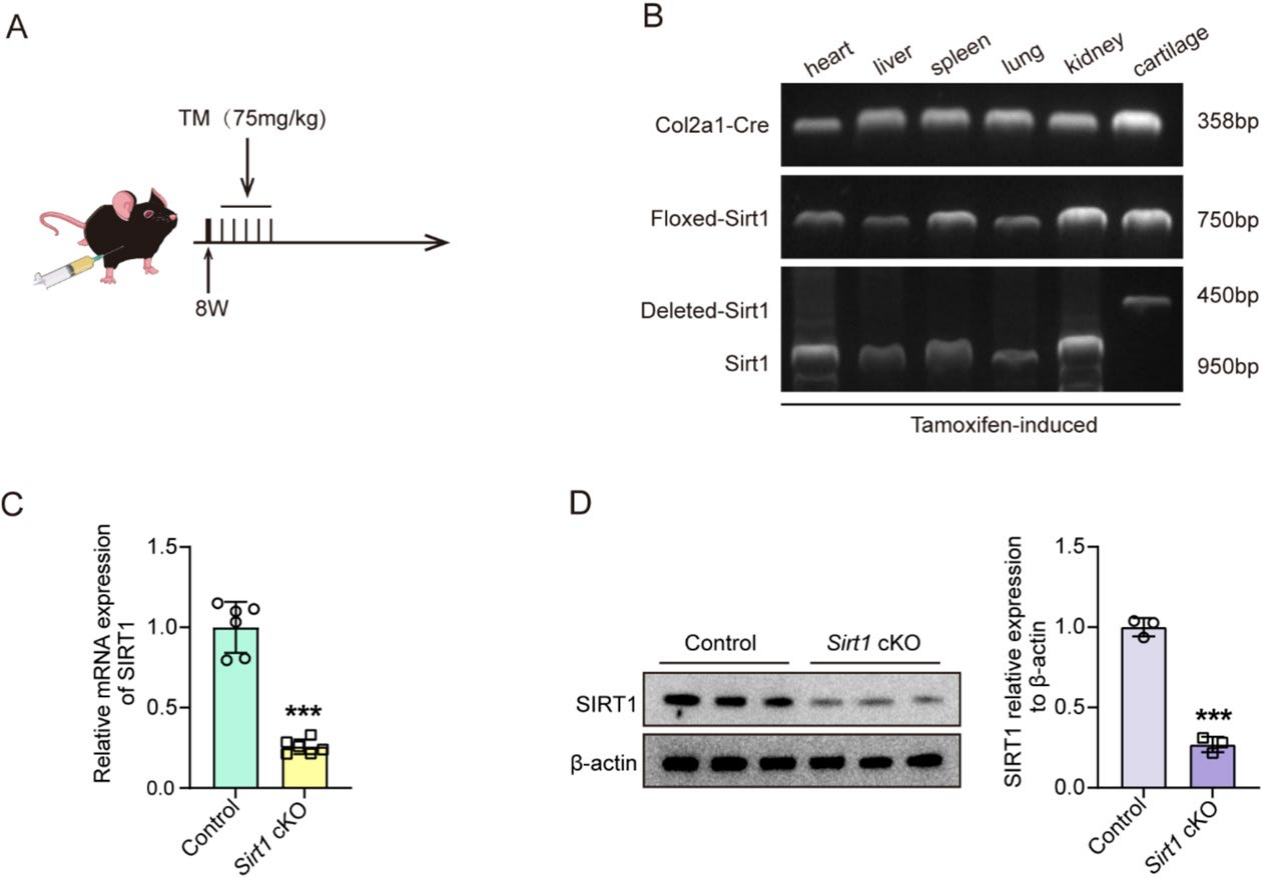
Verification of *Sirt1* cartilage-specific deletion in mice. **A** Schematic of TM administration. To induce *Sirt1* deletion, TM (75 mg/kg) was intraperitoneally injected for 5 consecutive days into 8-week-old mice. The *Sirt1*^*flox/flox*^ and Col2a1-CreERT; *Sirt1*^*flox/flox*^ mice received the identical amounts of TM to ensure consistency. **B** PCR analysis of gene expression of *Sirt1* and Col2a1-Cre in different tissues of mice. **C** RT-qPCR analysis of *Sirt1* mRNA expression in cartilage of Control and *Sirt1*cKO mice (n = 6/group). **D** Western blotting analysis of SIRT1 protein expression in cartilage of Control and *Sirt1*cKO mice. β-actin was used as an internal control. Right: quantitative analysis of the protein expression of SIRT1 in cartilage of control and *Sirt1*cKO mice (n = 3/group). Data are presented as mean ± SD, Student’s t test; ***P < 0.001

To confirm the protective effect of STS on cartilage is mediated by SIRT1, we used TM-inducible *Sirt1* cartilage-specific knockout mice (*Sirt1*cKO) by breeding *Sirt1*^*flox/flox*^ mice with Col2a1-cre/ERT mice (Fig. 8A). PCR results indicated that *Sirt1* was specifically deleted in the cartilage of *Sirt1*cKO mice, but not in the other five major tissues (heart, liver, spleen, lung, and kidney) (Fig. 8B). RT-qPCR and Western blotting results indicated that SIRT1 exhibited a 70% reduction in its mRNA and protein expression levels (Fig. 8C, D), validating the removal of *Sirt1* specifically in cartilage.

**Fig. 9:**
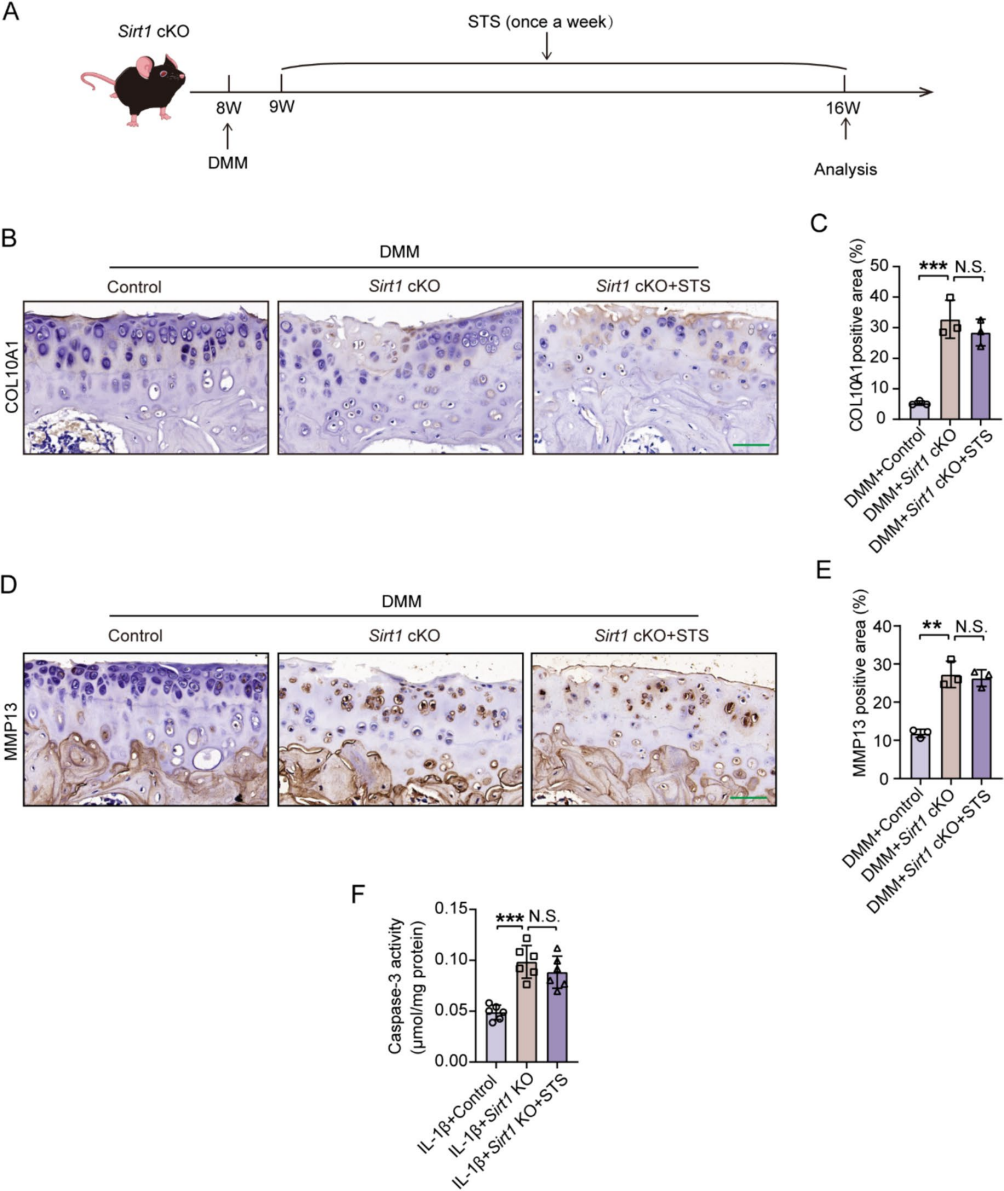
STS failed to alleviate OA progression in *Sirt1*cKO mice. **A** Schematic illustration of animal experimental design. Schematic diagram of *Sirt1*cKO mice subjected to DMM surgery in the right knee, followed by treatment via intra-articular administration of STS (45 μg). **B** Representative images of COL10A1 staining in the tibial articular cartilage of mice for each group (DMM + Control, DMM + *Sirt1*cKO, DMM + *Sirt1*cKO + STS). Bar = 50 μm. **C** Quantification of COL10A1 positive staining area in each group (n = 3/group). **D** Representative images of MMP13 staining in the tibial articular cartilage of mice for each group (DMM + Control, DMM + *Sirt1*cKO, DMM + *Sirt1*cKO + STS). Bar = 50 μm. **E** Quantification of MMP13 positive staining area in each group (n = 3/group). **F** Quantification of Caspase-3 activity from IL-1β-induced primary chondrocytes of each group (IL-1β + Control, IL-1β + *Sirt1*KO, IL-1β + *Sirt1*KO + STS) (n = 6/group). Data are presented as the mean ± SD, one-wayANOVA; N.S., no significant differences, **P < 0.01, ***P < 0.001

STS was then intraarticularly injected into the knee joint cavity of *Sirt1*cKO mice undergoing DMM surgery to evaluate the effects of STS on the expression of COL10A1 and MMP13, as well as Caspase3 (apoptosis) (Fig. 9A). Compared with the DMM surgery group, deletion of *Sirt1* in cartilage further increased COL10A1 and MMP13 expression, indicating that *Sirt1* deletion accelerates cartilage degeneration (Fig. 9B–E). However, STS treatment did not reverse the expression of these proteins (Fig. 9B–E). We further investigated the effects of STS on apoptosis in *Sirt1* KO primary chondrocytes induced by IL-1β. Deleting *Sirt1* increased Caspase-3 expression, accelerating apoptosis (Fig. 9F). However, STS treatment did not reduce Caspase-3 levels in these cells (Fig. 9F). These findings suggest that STS fails to play its protective role due to a lack of *Sirt1* in the cartilage of *Sirt1*cKO mice following DMM surgery and in *Sirt1* KO primary chondrocytes upon IL-1β induction.

**Fig. 10:**
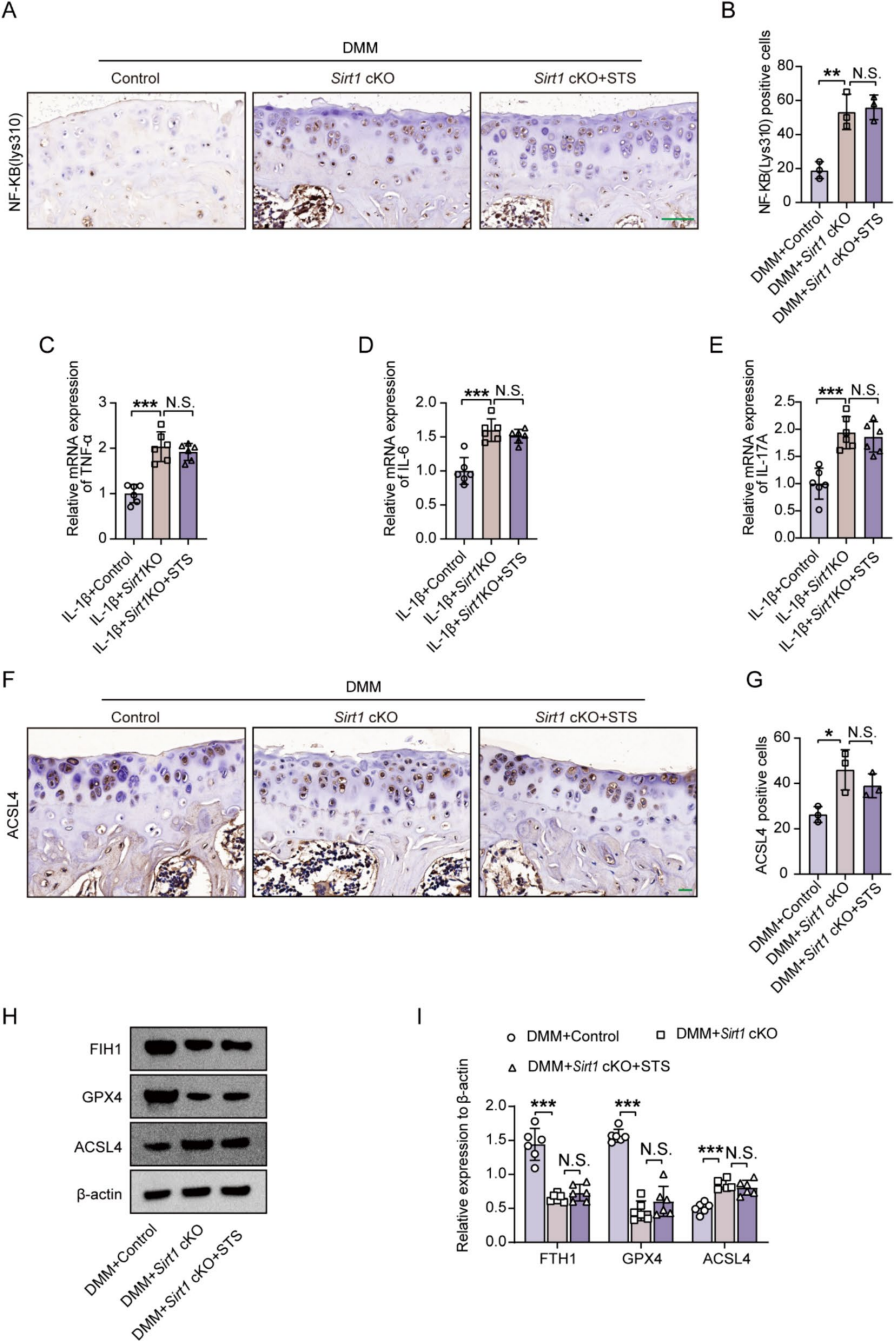
STS failed to alleviate the levels of NF-κB (Lys310), inflammation, and ferroptosis in *Sirt1*cKO mice. STS at a concentration of 45 μg or 100 μM was used in the study. **A** Representative images of NF-**κ**B (Lys310) staining in the tibial articular cartilage of mice for each group (DMM + Control, DMM + *Sirt1*cKO, DMM + *Sirt1*cKO + STS). Bar = 50 μm. **B** Quantification of NF-**κ**B (Lys310) positive staining cells in each group (n = 3/group). **C–E** The relative mRNA expression of TNF-α, IL-6, and IL-17A in the IL-1β-induced primary chondrocytes of each group (IL-1β + Control, IL-1β + *Sirt1*KO, IL-1β + *Sirt1*KO + STS) (n = 6/group). **F** Representative images of ACSL4 staining in the tibial articular cartilage of mice for each group (DMM + Control, DMM + *Sirt1*cKO, DMM + *Sirt1*cKO + STS). Bar = 20 μm. **G** Quantification of ACSL4 positive cells in each group (n = 3/group). **H** Protein levels of FTH1, GPX4, and ACSL4 from tibial cartilage of mice for each group (DMM + Control, DMM + *Sirt1*cKO, DMM + *Sirt1*cKO + STS). **I** Quantification of these proteins (n = 6/group). Data are presented as the mean ± SD, one-wayANOVA; N.S., no significant differences, *P < 0.05, **P < 0.01, ***P < 0.001

We further investigated whether the effects of STS on the deacetylation of NF-κB p65, inflammation, and ferroptosis were dependent on SIRT1. Immunohistochemical staining results indicated that *Sirt1* deletion promoted higher expression of NF-κB p65 (Lys310) in the cartilage of *Sirt1*cKO mice under DMM surgery, but STS failed to rescue such effect (Fig. 10A, B). Meanwhile, ablation of *Sirt1* also induced higher expression of inflammation factors (TNF-α, IL-6, and IL-17A) in IL-1β-induced *Sirt1* KO primary chondrocytes, but STS treatment had no effects on the expression of these inflammatory factors (Fig. 10C–E). Moreover, ablation of *Sirt1* also induced the protein expression of ACSL4, FTH1, and GPX4, markers of ferroptosis, in the cartilage of *Sirt1*cKO mice under DMM surgery, however, STS treatment was ineffective in regulating these protein expressions (Fig. 10F-I).

**Fig. 11:**
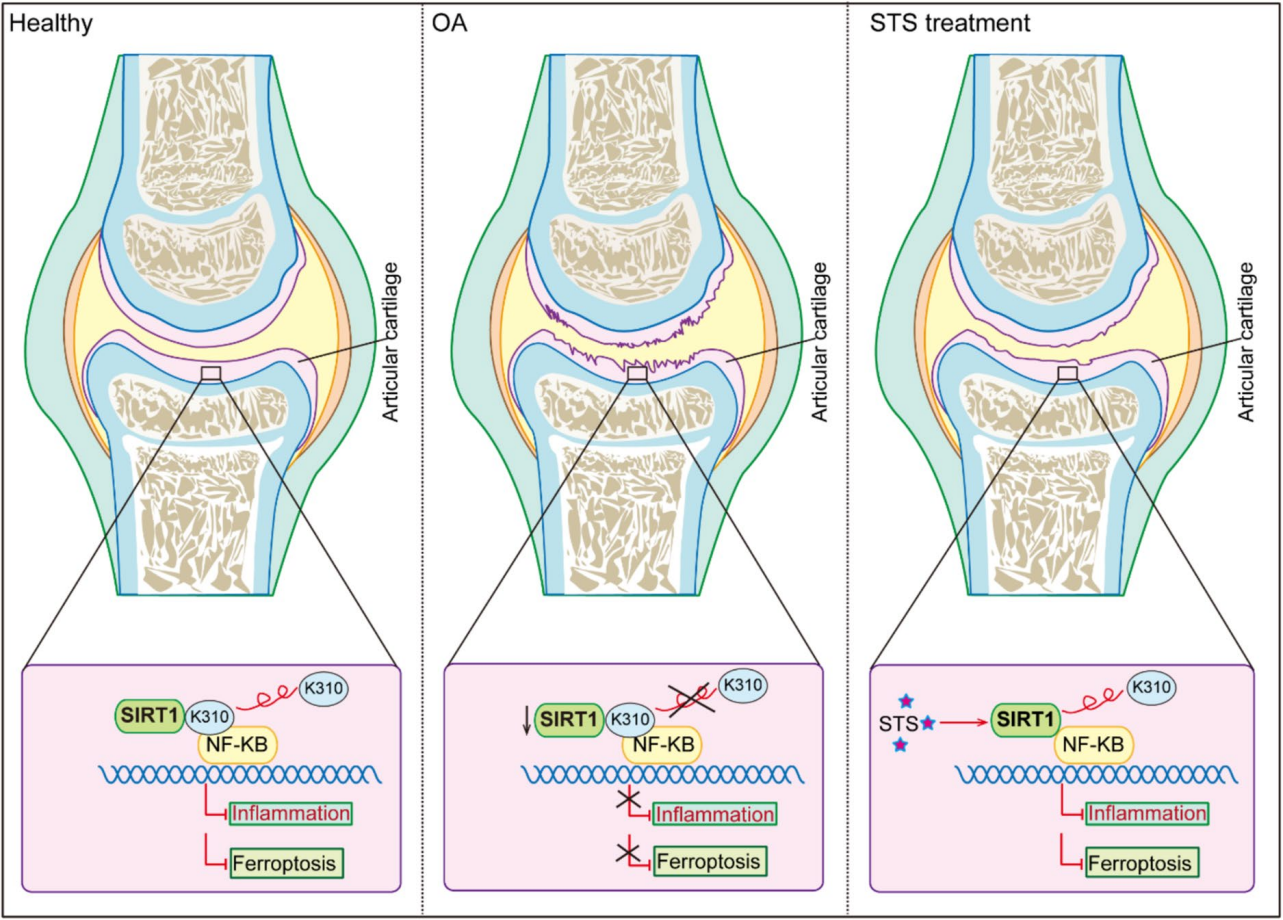
Working model shows that STS alleviates OA by activating SIRT1 deacetylation of NF-κB p65 (Lys310) and inhibiting NF-κB-driven inflammation and ferroptosis

Altogether, our results indicate that SIRT1 expression is upregulated by STS, which in turn enhances the deacetylation of NF-κB p65. This deacetylated NF-κB p65 subsequently suppresses downstream inflammatory responses and ferroptosis (Fig. 11).

## Discussion

In our study, we evaluated the effects of STS in treatment of cartilage degeneration using an OA mouse model. Our results indicate that STS alleviates cartilage degradation by activation of SIRT1, and in turn promotes deacetylation of NF-κB p65, and thereby inhibiting inflammation and ferroptosis.

We found STS alleviated OA progression. DMM model was employed in C57 mice to evaluate the effects of STS on OA. This model is widely preferred over other OA models, such as instability models, spontaneous models, and surgical OA models, due to its high reproducibility and ability to closely mimic the gradual progression of human OA from mild to moderate stages [42]. Our present study revealed that the DMM surgery effectively induced a phenotype akin to OA within the knee joint's cartilage in mice. Pharmaceutical agents for OA management can be administered through both oral and intra-articular routes [43]. However, the delivery of drugs to chondrocytes is hindered by the absence of vascular supply in cartilage [44, 45]. Thus, the diffusion of numerous large therapeutic molecules, particularly biologics, from synovial capillaries (the nearest vasculature) into the joint space is limited. Alternatively, intra-articular injection has gained popularity as a viable method for drug delivery [46]. Therefore, STS was intra-articularly injected to alleviate the cartilage damage induced by DMM surgery in our study. Various risk factors contribute to OA through its associated endpoint phenotypes, especially cartilage damage. These risk factors operate via a shared pathogenesis characterized by enhanced chondrocyte hypertrophy, increased expression of cartilage catabolic enzymes and apoptosis [47]. We found that STS inhibited the levels of chondrocyte hypertrophic marker (COL10A1) and catabolic enzyme (MMP13), as well as apoptosis in chondrocytes, thus alleviating OA. Our results indicate that STS may be a potential therapeutic agent for OA.

When STS is utilized for intra-articular injection in humans, several critical factors, such as dosage conversion, variations in drug uptake, diffusion, and retention etc., must be considered given the differences in cartilage thickness [48–53]. Of note, to achieve optimal drug-release and extend pharmaceutical half-life, developing new sustained-release drug delivery systems for STS in OA treatment is crucial [54]. This includes the development of hydrogels [55, 56], nanoparticles [57], cell membrane biomimetic nanoparticles [58], and nanocomposite hydrogels [59].

The disorganization of joint structure is largely influenced by alterations in molecular and signaling pathways associated with aging [60]. SIRT1, originally thought to slow down the aging process, has been found to play a protective role in maintaining cartilage homeostasis, as evidenced by clinical, animal model, and cellular studies [33]. The serum NT/CT SIRT1 ratio can serve as an indicator for early OA and chondrosenescence [61]. *Sirt1* null, heterozygous, and homozygous mice exhibited OA phenotypes [62–64]. Correspondingly, SIRT1 knock-in mice showed delayed OA phenotypes [65]. Moreover, mice with chondrocyte-specific deletion of *Sirt1* (Col2a1-Cre) exhibited accelerated OA progression after DMM surgery [28]. In line with these findings, our research group utilized TM-inducible cartilage-specific *Sirt1* deletion mice (Col2a1-CreERT; *Sirt1*^−/−^) and also observed that *Sirt1* deletion led to chondrocyte hypertrophy and apoptosis, resulting in spontaneous OA development [30, 31]. Additional cellular and molecular investigations have demonstrated that SIRT1 plays a protective role in OA by upregulating anabolic genes and downregulating catabolic genes [33]. Resveratrol, an early identified SIRT1 activator, has shown efficacy in alleviating the onset and progression of OA [32]. In addition, compounds such as curcumin, quercetin, and safflower yellow have been found to activate SIRT1 expression, thereby reducing articular cartilage degradation and inhibiting chondrocyte inflammation and apoptosis [66–68]. Recent studies have demonstrated that STS had potential to promote the SIRT1 expression, thereby providing relief for various diseases, including non-alcoholic fatty liver disease, chronic obstructive pulmonary disease, and cardiovascular disease [20, 34, 69]. Consistent with these findings, our experimental results suggest that STS can promote SIRT1 expression, thus alleviating OA. This conclusion was further supported by the lack of efficacy of STS treatment on TM-inducible cartilage-specific *Sirt1* deletion mice (*Sirt1*cKO) with OA. Therefore, our results indicate that STS may effectively alleviate OA in mice by promoting SIRT1.

Previous research has shown that SIRT1 deacetylates NF-κB p65 to inhibit NF-κB signaling, exhibiting protective effects on OA [28]. A recent study has demonstrated that STS upregulates circ-*Sirt1* and subsequently inhibites the nuclear entry of NF-κB in LPS-treated RAW264.7 cells [40]. Similarly, our present results indicate that STS promotes the deacetylation of NF-κB p65 on Lys310 by SIRT1. Seven acetylation sites, including Lys310, have been identified in the NF-κB p65 subunit [70, 71]. Acetylation at Lys310 is essential for the full transcriptional activity of NF-κB p65 [72, 73]. Notably, SIRT1 has been demonstrated to suppress NF-κB transcriptional activity through the direct deacetylation of the NF-κB p65 subunit at lysine 310, while maintaining the acetylation status of other lysine residues [74]. This regulatory mechanism is fundamental in macrophages [75], epithelial cells [76], and human dermal fibroblasts [77]. In chondrocytes, SIRT1 activation or overexpression has been observed to alleviate the progression of experimental OA, whereas inhibition or cartilage-specific deletion of SIRT1 exacerbates OA development by modulating the acetylation level of NF-κB p65 at lysine 310 [28, 78–80]. Consequently, our study focused on examining the impact of STS on the modulation of SIRT1's effects on the acetylation status of lysine 310 in NF-κB p65. NF-κB facilitates the transcription process in the genes responsible for inflammatory mediators, including IL-1β, TNF-α, IL-6, and IL-17A [81, 82]. The proinflammatory cytokines, IL-1β, TNF-α, and IL-17A, have been observed to decrease the expression of anabolic factors and increase the expression of catabolic factors, leading to chondrocyte hypertrophy, dedifferentiation, and apoptosis [83]. A recent investigation has provided evidence of STS's ability to alleviate inflammation and apoptosis induced by IL-1β in meniscal fibrochondrocytes [26]. In addition, STS has been observed to decrease the production of IL-1β and IL-6 in TNF-α-treated RAhuman fibroblast-like synoviocytes [27]. Thus, we propose that the inflammatory mediators may be the downstream effectors of STS/SIRT1/ NF-κB p65 signaling in chondrocytes. As expected, we observed STS treatment effectively mitigated the expression of SIRT1, NF-κB p65 (Lys310), and inflammatory cytokines (IL-1β, TNF-α, IL-6, and IL-17A) in OA mice and primary chondrocytes. Therefore, it is reasonable to conclude that STS activates SIRT1 and promotes SIRT1-mediated deacetylation of NF-κB p65, the deacetylated NF-κB p65 then inhibits the gene transcription of inflammatory cytokines. These findings suggested that STS exertes anti-inflammatory effects by modulating the SIRT1/ NF-κB p65 pathway, thereby protecting against OA.

Ferroptosis is a form of cell death reliant on iron and lipid peroxidation, characterized by iron buildup, impaired lipid repair, and membrane damage [84, 85]. It is increasingly recognized for its role in chondrocyte apoptosis and matrix degradation, which contribute to OA. Research shows a strong link between ferroptosis-related pathways and chondrocyte dysfunction. For example, GPX7 overexpression may protect chondrocytes by modulating ferroptosis, as it inhibits IL-1β-induced inflammation, extracellular matrix degradation, and chondrocyte apoptosis [86]. Traditional Chinese medicine compounds, such as paeoniflorin, may slow OA progression by targeting ACSL4 to reduce IL-1β/ferric ammonium citrate-induced damage, including cell apoptosis, inflammation, extracellular matrix degradation, and ferroptotic markers [87]. Additionally, quercetin reduces ferroptosis and IL-1β-induced chondrocyte apoptosis by activating the AMPK/NRF2/GPX4 pathway [88]. Ferroptosis-related genes such as LPCAT3 and PGD may serve as diagnostic markers, and targeting ferroptosis could provide therapeutic benefits for OA [89]. Recent studies indicate that NF-κB promotes ferroptosis across various diseases by influencing iron metabolism, oxidative stress, and inflammation [90–93]. The induction of ferroptosis by NF-κB is also observed in OA. In a study on sodium iodoacetate-induced OA rat models, inhibiting the NF-κB p65 pathway reduced chondrocyte ferroptosis and matrix degradation. Moderate mechanical stress activated the Nrf2 antioxidant system, which suppressed NF-κB p65 signaling and regulated ferroptosis-related genes like GPX4 and SLC7A11, protecting chondrocytes from IL-1β-induced damage. However, overexpression of NF-κB p65 negated these protective effects, highlighting its key role in OA-related ferroptosis [94]. Additionally, modulation of Nrf2 signaling can inhibit NF-κB activation, reducing inflammation and protecting chondrocytes from ferroptosis [95]. Activation of FOXO3 also impedes ferroptosis via the NF-κB/MAPK pathway, thereby slowing OA progression [96]. Similarly, our study indicates that NF-κB induces ferroptosis in OA. These findings identify NF-κB and ferroptosis as crucial factors in OA pathogenesis and promising therapeutic targets. Researchers are exploring new drugs, with natural compounds like pantothenic acid and quercetin showing potential by inhibiting ferroptosis through the NF-κB pathway [97, 98]. Other compounds are being studied for enhancing chondrocyte function and slowing OA progression by targeting NF-κB and ferroptosis [99]. Innovative approaches, such as pH-responsive lipid nanoparticles, aim to target OA lesions and improve the chondrocyte environment by activating mitophagy and inhibiting ferroptosis [100]. Similarly, our study found that STS protect against OA by modulating the SIRT1/NF-κB-induced ferroptosis pathway.

In our study, STS promotes SIRT1, which deacetylates NF-κB p65 to block NF-κB-driven cartilage inflammation and ferroptosis, and in turn protects chondrocytes from apoptosis, thus maintaining cartilage homeostasis as well as alleviating the progression of cartilage degeneration.

## Conclusions

STS activated SIRT1, leading to the deacetylation of NF-κB p65 at lysine 310 via SIRT1’s enzymatic activity. This modification significantly reduced NF-κB’s transcriptional activity, especially in regulating genes linked to inflammation and ferroptosis pathways.

## Supplementary Information

Additional file 1.

Additional file 2.

## Data Availability

The data supporting the findings of this study are available within the article and the supplementary materials.

## Abbreviations

OA: Osteoarthritis
STS: Sodium tanshinone IIA sulfonate
SIRT1: Silent information regulator 1
COL10A1: Collagen type X alpha 1
MMP13: Matrix metalloproteinase-13
COL2A1: Collagen type II alpha 1
LPO: Lipid peroxide
MDA: Malondialdehyde
GSH: Glutathione
4-HNE: 4-Hydroxy-2-nonenal
ACSL4: Acyl-CoA synthetase long-chain family member 4
FTH1: Ferritin heavy chain 1
GPX4: Glutathione peroxidase 4
DMM: Destabilization of the medial meniscus
OARSI: Osteoarthritis Research Society International
CFDA: The China State Food and Drug Administration
TM: Tamoxifen
cKO: Conditional knockout
IL-1β: Interleukin-1β
RT-qPCR: Real-time quantitative PCR
ELISA: Enzyme-linked immunosorbent assay
IP: Immunoprecipitation
ROS: Reactive oxygen species
HE: Hematoxylin–eosin
K: Lysine
SD: Standard deviation
ANOVA: A one-way analysis of variance
