# Supplementary material for: Sodium tanshinone IIA sulfonate alleviates osteoarthritis through targeting SIRT1
Source: Chin Med. 2025 Sep 1;20:142. doi: 10.1186/s13020-025-01166-2 (PMC12400743; doi:10.1186/s13020-025-01166-2)
Supplement: Supplementary file 1 — Additional file 1. [file 13020_2025_1166_MOESM1_ESM.docx]

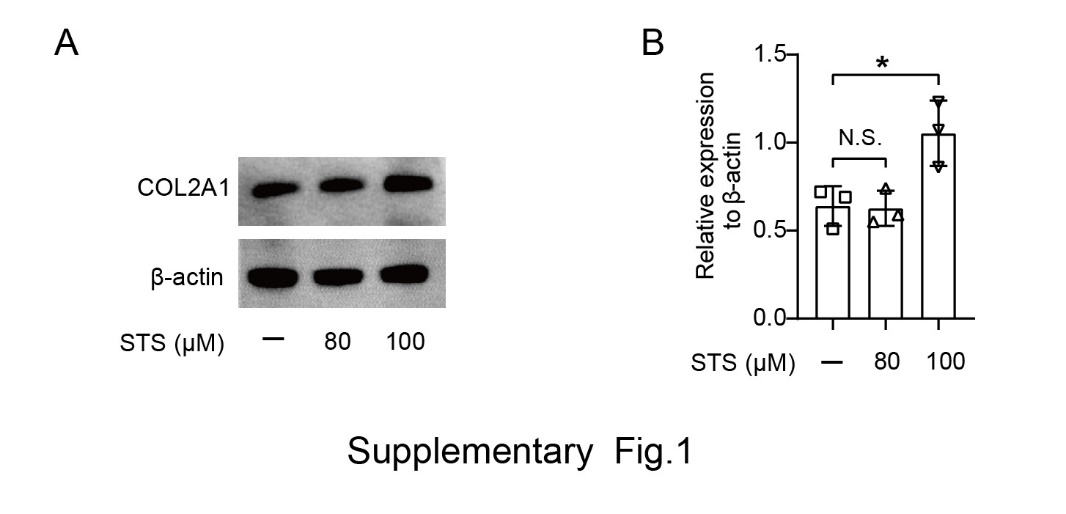


**Supplementary Figure 1. The effects of STS on protein level of COL2A1. (A)** Protein expression of COL2A1 in the primary chondrocytes following treatment with two concentrations of STS (80μM and 100μM). **(B)** Quantification of COL2A1 protein expression in the primary chondrocytes following treatment with two concentrations of STS (80μM and 100μM) (n=3/group). Data are means ± SD. One way-ANOVA for three groups; N.S., no significant differences, **P* < 0.05.


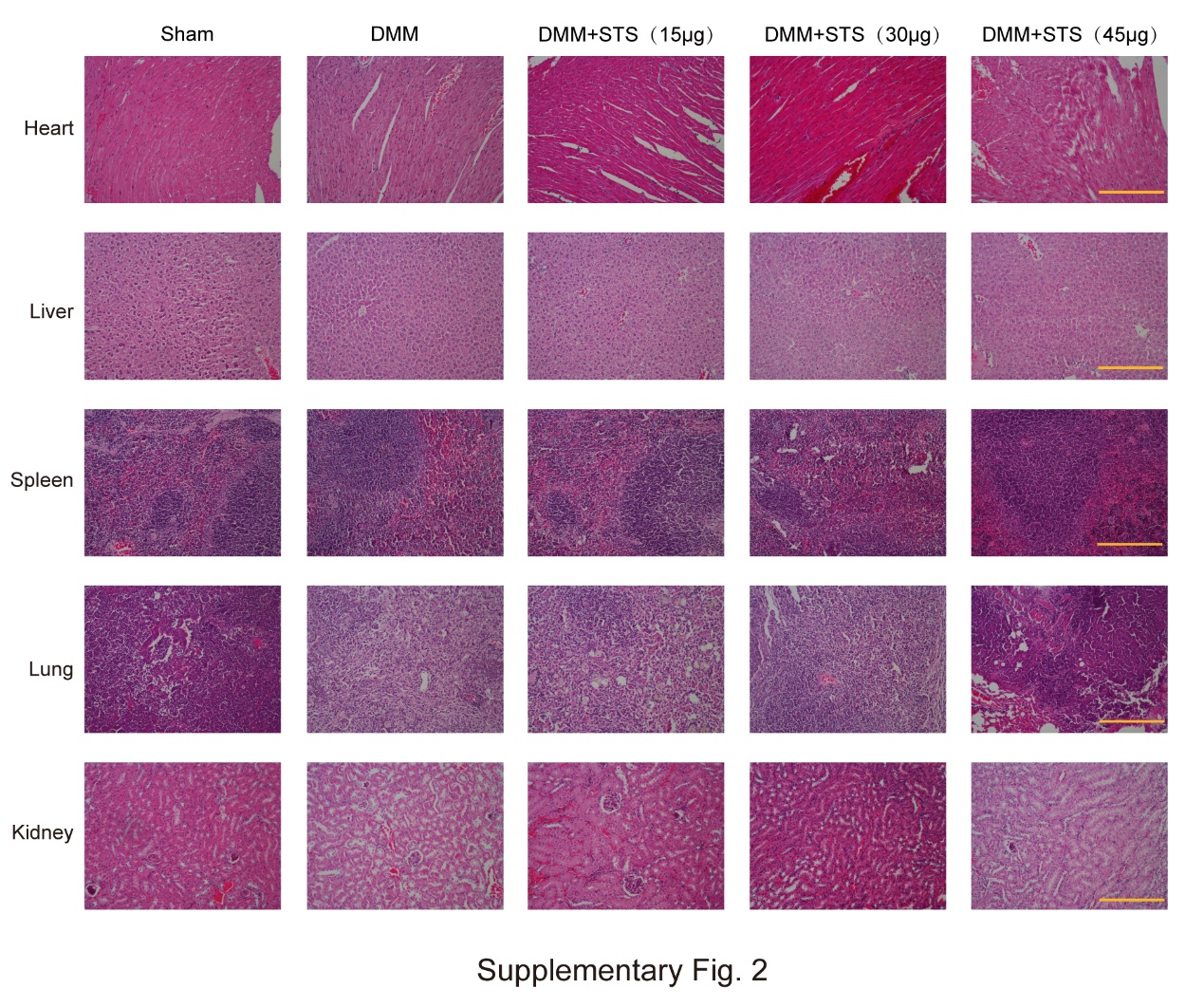


**Supplementary Figure 2.** **The structure of major organs after STS intraarticular administration in each group.** Bar=100 μm.
