# Supplementary material for: Sodium tanshinone IIA sulfonate alleviates osteoarthritis through targeting SIRT1
Source: Chin Med. 2025 Sep 1;20:142. doi: 10.1186/s13020-025-01166-2 (PMC12400743; doi:10.1186/s13020-025-01166-2)
Supplement: Supplementary file 2 — Additional file 2. [file 13020_2025_1166_MOESM2_ESM.docx]

**Supplementary** **Table 1.** The primer sequences of the RT-qPCR reaction.

| **Name** | **Sequences** |  |  |
| --- | --- | --- | --- |
| β-actin | Forward: 5’-TTCCAGCCTTCCTTCTTG-3’ |  |  |
|  | Reverse: 5’- GGAGCCAGAGCAGTAATC-3’ |  |  |
| Col10a1 | Forward: 5’-CCGCTTGTCAGTGCTAAC-3’ |  |  |
|  | Reverse: 5’-GTAATGCTGCTGCCTATTGT-3’ |  |  |
| Mmp13 | Forward: 5’-ACAGTTGACAGGCTCCGAGAA-3’ |  |  |
|  | Reverse: 5’-CACATCAGGCACTCCACATCTTG-3 |  |  |
| IL-6 | Forward: 5’-ACCTGTCTATACCACTTC-3’ |  |  |
|  | Reverse: 5’-GCATCATCGTTGTTCATA-3’ |  |  |
| IL-17A | Forward: 5’- CTCTCCACCGCAATGAAG-3’ |  |  |
|  | Reverse: 5’- TCAGGACCAGGATCTCTTG-3’ |  |  |
| TNF-α | Forward: 5’-CCTATGTCTCAGCCTCTT-3’ |  |  |
|  | Reverse: 5’-GAACTTCTCATCCCTTTGG-3’ |  |  |
| Sirt1 | Forward: 5’-CCTGTGGGATTCCTGACTTC-3’ |  |  |
|  | Reverse: 5’-ATACTCAATACAAACATGGCTTG-3’ |  |  |

**Supplementary Table 2.** Primer sequences for genotyping.

| **Name** | **Sequences** |
| --- | --- |
| Flox-Sirt1 | 7909, 5’-GGTTGACTTAGGTCTTGTCTG-3’ |
|  | 7911, 5’-AGGCGGATTTCTGAGTTC GA-3’ |
|  | 7912, 5’-CGTCCCTTGTAATGTTTCCC-3’ |
| Col2a1-CreER^T^ | Forward, 5’-CACTGCGGGCTCTACTTCAT-3’ |
|  | Reverse, 5’ -ACCAGCAGCACTTTTGGA AG-3’ |
